# Redundancy analysis allows improved detection of methylation changes in large genomic regions

**DOI:** 10.1186/s12859-017-1986-0

**Published:** 2017-12-14

**Authors:** Carlos Ruiz-Arenas, Juan R. González

**Affiliations:** 1 ISGlobal, Centre for Research in Environmental Epidemiology (CREAL), Barcelona, Spain; 2 Universitat Pompeu Fabra (UPF), Barcelona, Spain; 3 CIBER Epidemiología y Salud Pública (CIBERESP), Barcelona, Spain

**Keywords:** DNA methylation, Region analysis, Microarray, Epigenomics, Gene expression

## Abstract

**Background:**

DNA methylation is an epigenetic process that regulates gene expression. Methylation can be modified by environmental exposures and changes in the methylation patterns have been associated with diseases. Methylation microarrays measure methylation levels at more than 450,000 CpGs in a single experiment, and the most common analysis strategy is to perform a single probe analysis to find methylation probes associated with the outcome of interest. However, methylation changes usually occur at the regional level: for example, genomic structural variants can affect methylation patterns in regions up to several megabases in length. Existing DMR methods provide lists of Differentially Methylated Regions (DMRs) of up to only few kilobases in length, and cannot check if a target region is differentially methylated. Therefore, these methods are not suitable to evaluate methylation changes in large regions. To address these limitations, we developed a new DMR approach based on redundancy analysis (RDA) that assesses whether a target region is differentially methylated.

**Results:**

Using simulated and real datasets, we compared our approach to three common DMR detection methods (Bumphunter, blockFinder, and DMRcate). We found that Bumphunter underestimated methylation changes and blockFinder showed poor performance. DMRcate showed poor power in the simulated datasets and low specificity in the real data analysis. Our method showed very high performance in all simulation settings, even with small sample sizes and subtle methylation changes, while controlling type I error. Other advantages of our method are: 1) it estimates the degree of association between the DMR and the outcome; 2) it can analyze a targeted or region of interest; and 3) it can evaluate the simultaneous effects of different variables. The proposed methodology is implemented in MEAL, a Bioconductor package designed to facilitate the analysis of methylation data.

**Conclusions:**

We propose a multivariate approach to decipher whether an outcome of interest alters the methylation pattern of a region of interest. The method is designed to analyze large target genomic regions and outperforms the three most popular methods for detecting DMRs. Our method can evaluate factors with more than two levels or the simultaneous effect of more than one continuous variable, which is not possible with the state-of-the-art methods.

**Electronic supplementary material:**

The online version of this article (10.1186/s12859-017-1986-0) contains supplementary material, which is available to authorized users.

## Background

DNA methylation is an epigenetic mechanism where a methyl group is added to cytosines placed in CG dinucleotides (CpGs). This process regulates cellular gene expression and is responsible for biological processes such as X chromosome inactivation. Disruption of the methylation pattern can lead to diseases such as cancer [1, 2] or diabetes [3, 4]. DNA methylation can be modified by environmental exposures (e.g. smoking [5–7]) so it is becoming a common tool in epidemiological studies.

DNA methylation microarrays allow performing a genome-wide evaluation of the methylation status. The analysis of these microarrays is comparable to the analysis of gene expression microarrays. The current standard analysis for differential gene expression consists of performing a linear regression of each expression probe against a variable of interest and any relevant covariables. To test the significance, an empirical Bayes approach using the variance of all genes is commonly used [8]. The result of this analysis is a list of expression probes that are most strongly associated with the outcome of interest. This method was adapted for use in methylation studies, where methylation values of individual CpG sites are regressed against the variable of interest.

However, some authors suggest that methylation changes usually occur at regional level [9, 10] and, in practical terms, this involves detecting groups of consecutive methylation probes that are associated with the outcome (Differentially Methylated Regions, DMRs). A number of methods have been implemented in R for this type of analyses: Bumphunter [11], DMRcate [12], Probe Lasso [13], IMA [14] and MethyAnalysis [15]. We will focus on Bumphunter and DMRcate as these are the most popular methods and are implemented in several methylation analysis pipelines (e.g. Champ package [16]). Both methods are based on linear regression models, and use various statistical techniques to scan the genome for groups of probes associated with the variable of interest, and provide lists of small DMRs (<2Kb).

Several authors have suggested that some factors (e.g. genomic structural variants) can modify the methylation patterns of large regions, between 100 Kb and several Mbs [17–20]. Furthermore, it has been proposed that in cervical cancer changes in the methylation pattern of a large region can have a similar effect to the deletion of that region [20]. Although Bumphunter and DMRcate are powerful enough to detect changes in methylation patterns in small regions [21], they have limited ability to detect large DMRs. Both methods define candidate DMRs based on the observed data, i.e. regions are considered depending on the observed effects of consecutive CpGs. However, these packages are not able to test whether or not a target region is differentially methylated. Bumphunter was designed to detect DMRs where methylation changes are in the same direction, i.e. most of the CpGs in the region must be hypo- or hyper-methylated sites. While this assumption may hold in small regions, large regions are likely to contain probes that are positively and negatively associated with the outcome of interest. Consequently, averaging the effect of the entire region may provide a signal close to zero because the effects are compensated. DMRcate parameters can be modified to detect large DMRs, although the accuracy of the method in these conditions has not been properly tested. Another of DMRcate’s limitations is that it does not provide a measure of the statistical significance of the association. Subsequently, blockFinder, an adaptation of Bumphunter [22], was designed to find big DMRs, although it has some drawbacks: 1) it also requires that all changes are in the same direction; and 2) it only considers open sea probes, and thus uses only a small fraction of all methylation probes. A common limitation of these three methods is that they can only evaluate the effect of a single continuous or a dichotomous factor. Therefore, it is not possible to directly assess the combined effect of two variables or a categorical variable with more than two levels.

To tackle these limitations, we propose a method for assessing whether a target region of any size is a DMR. Our method can detect these regions, even in the presence of epigenetic changes in different directions. Analyzing DMRs can be seen as an extension of considering more than one CpG as the outcome. Thus, our method is based on a multivariate approach called redundancy analysis (RDA), which extends linear regression to multivariate outcomes [23]. For the epigenomic data in our study, we will consider methylation values of the region as a multivariate outcome and the factor (i.e. case/control, sex, age…) as the explanatory variable. Our new DMR method is implemented in MEAL [24], a Bioconductor package for analyzing methylation and expression data. MEAL includes functions for performing not only RDA but also other analyses such as single probe analysis, allowing the user to adjust for covariates such as surrogate variables that control for batch effects or cell composition. To facilitate data visualization, the package also contains state-of-the-art plotting functions, such as a Manhattan or a Volcano plot.

In this paper, we demonstrate the advantages of using RDA to perform DMR analyses, emphasizing situations where methylation changes are produced in large genomic regions. We compare its performance with three existing approaches for detecting DMRs (Bumphunter, blockFinder, and DMRcate) using simulated and real datasets.

## Implementation

RDA can be thought of as a method for performing a linear regression between a multivariate outcome (matrix Y, e.g. multiple CpGs in a region) and a table of regressors (matrix X, e.g. case/control, sex, age…). RDA has two steps. First, a multiple linear regression between each variable of matrix Y and all variables of matrix X is run (Additional file 1: Figure S1). This step results in a matrix with fitted values and a matrix with the residuals. The second step is running a Principal Components Analysis to fitted and residuals tables. PCA components of the fitted matrix are also called RDA components. As a result, the main assumptions of the data are linearity between the variables of matrix Y and variables of matrix X and variance homogeneity of each dataset. RDA analysis for methylation data has been implemented in a function called *runRDA*.

RDA returns two statistics that are useful for measuring the degree of association between our grouping factor variable and the methylation in a DMR: the R-squared (R^2^), and the RDA components. R^2^ measures the percentage of the variability observed in the CpGs in the region of interest that is explained by the variable of interest (e.g. case/control, quantitative trait, multi-class variable, etc.). The RDA components are useful for visualizing the results and seeing how, in the case of categorical outcomes, individuals cluster on CpGs variables.

The significance of the association between the factor and the region of interest is assessed using two approaches. In the first approach, we use a permutation test implemented in the function *permutest* of vegan R package [25]. This test returns a *p*-value indicating whether RDA components are significantly associated with the variable that is defining the different levels of groups of our variable of interest. We have implemented another approach that is providing a measure (*p*-value) of the evidence against the probability of finding a random region in the genome with an R^2^ greater than or equal to that obtained in our region of interest. It is expected that, if our target region is not associated with our variable of interest, the R^2^ of such association obtained from RDA would be lower than the R^2^ observed in any other region of the genome having the same number of CpGs as in our target region. This approach is implemented in the function *computeRDAR2* of MEAL.

Our *runRDA* function implemented in MEAL package [24] accepts as input several types of Bioconductor objects such as *GenomicRatioSet* or *GenomicRanges*, facilitating the use of our new method. *runRDA* relies on the RDA implementation of vegan R package. Biplots can be created to help RDA visualization and interpretation. The MEAL package also includes a number of functions to perform methylation data analysis including DMP, DMR and variance comparison among groups. Adjustment for covariates or/and surrogate variables (SVA) can also be performed [26]. State-of-the-art plots can also be created with MEAL.

## Results

### Simulation

We compared our RDA method against three well-known methods (Bumphunter, blockFinder and DMRcate) in simulated datasets. A Beta distribution was used to simulate methylation data (i.e. beta values). The simulation was performed by simulating 8432 CpGs belonging to chromosome 22. Parameters of the beta distribution were based on methylation data obtained from real data belonging to a birth cohort study (*n* = 396). Notice that our RDA method transforms these data into M-values to hold normality assumption. In each simulated dataset, we introduced a DMR by generating differentially methylated CpGs for two groups (groups 1 and 2). There were DMRs of four different sizes (500Kb, 300Kb, 100 Kb, and 50Kb) and datasets of three different sample sizes (10, 40, and 100). We simulated scenarios for three different effect sizes (difference in mean methylation: 0.3, 0.1 and 0.05), and two different percentages of differentially methylated probes (30% and 10%) within the region. We simulated 200 datasets of each combination of DMR size, sample size, and scenario. Additionally, we have evaluated BlockFinder, DMRcate and RDA methods in regions randomly in order to estimate the number of false positive results.

Bumphunter and BlockFinder was ran with 1000 permutations to determine the bumps’/blocks *p*-value and setting the minimum effect size to consider a probe in a bump/block to 0.05. RDA was run with 10,000 permutations to compute *p*-values. Next, we describe the main simulation results based on the results obtained from *n* = 40 (unless stated otherwise).

Bumphunter included all true differentially methylated probes in bumps when the effect size was equal to 0.3 (Table 1), while in the other scenarios, Bumphunter’s bumps only contained a small proportion of the simulated DMPs. As expected, Bumphunter’s performance decreased as sample size decreased (*n* = 10) (Additional file 1: Table S1). With a sample size of 100, Bumphunter’s performance increased but it still only detected a small fraction of the real changes for effect sizes smaller than 0.3 (Additional file 1: Table S2).

**Table 1:** Analysis of methylation regions using current methods (40 samples)

Region size	Sim. DMP %	Diff. Means	CpGs in Bumps (%)	R^2^ (sd)	
				Target region	Random region
500Kb	30	0.3	28.10	0.647 (0.109)	0.026 (0.010)
	30	0.1	3.23	0.430 (0.098)	0.025 (0.010)
	30	0.05	0.21	0.346 (0.124)	0.026 (0.010)
	10	0.3	9.16	0.442 (0.075)	0.024 (0.008)
	10	0.1	0.45	0.277 (0.101)	0.026 (0.009)
	10	0.05	0.02	0.205 (0.135)	0.025 (0.008)
300Kb	30	0.3	28.10	0.674 (0.113)	0.026 (0.013)
	30	0.1	3.76	0.417 (0.083)	0.025 (0.008)
	30	0.05	0.11	0.332 (0.127)	0.026 (0.011)
	10	0.3	8.80	0.446 (0.084)	0.026 (0.009)
	10	0.1	0.30	0.278 (0.100)	0.026 (0.010)
	10	0.05	0.02	0.178 (0.112)	0.026 (0.011)
100Kb	30	0.3	27.80	0.682 (0.125)	0.025 (0.011)
	30	0.1	3.58	0.430 (0.103)	0.026 (0.011)
	30	0.05	0.15	0.317 (0.122)	0.026 (0.011)
	10	0.3	8.72	0.453 (0.104)	0.027 (0.014)
	10	0.1	0.35	0.249 (0.112)	0.025 (0.010)
	10	0.05	0.00	0.171 (0.123)	0.025 (0.011)
50Kb	30	0.3	27.70	0.705 (0.120)	0.027 (0.012)
	30	0.1	3.93	0.426 (0.096)	0.027 (0.012)
	30	0.05	0.10	0.308 (0.096)	0.026 (0.010)
	10	0.3	8.60	0.442 (0.120)	0.028 (0.017)
	10	0.1	0.42	0.250 (0.100)	0.026 (0.013)
	10	0.05	0.03	0.149 (0.090)	0.028 (0.015)

Values represent the mean of the 200 simulations. *Sim. DMP %*, percentage of DMPs introduced in the simulation; *Diff. Means*, Difference in mean methylation between groups A and B; *CpGs in Bumps* (%), proportion of CpGs in the modified region that are within a bump with FDR < 0.05. *R* ^*2*^, R^2^ estimate of RDA model; *Target region*, region that includes our simulated DMPs; *Random region*, region without any of the simulated DMPs

We ran DMRcate using two different combinations of the lambda and C parameters to optimize its ability to detect large DMRs. Lambda represents the minimum distance between two significant DMPs for them to be assigned to two different DMRs. C controls the standard deviation of the Gaussian kernel used to smooth the overall signal in the region. In the first combination, we set lambda as equal to the size of our target region. C had the default value, such that the standard deviation of the Gaussian kernel was greatly increased, and we called this scenario *extended smoothing*. In the second combination, we changed C to the default standard deviation of the Gaussian kernel, and we called this scenario *standard smoothing*. While we obtained higher recall using the extended smoothing (Additional file 1: Figure S2), these DMRs had Stouffer *p*-values close to 1 (Additional file 1: Figure S3). This means that these DMRs are heterogeneous and contain many CpGs non-differentially methylated, suggesting that DMRs are too wide. Consequently, we decided to use the standard smoothing in the comparisons. As expected, if we required that a DMRcate’s DMR comprised a higher proportion of the target region, the power decreased (Additional file 1: Figure S4). These results were very similar for sample sizes of 10 and 100 (Additional file 1: Figures S5-S10).

**Fig. 1:**
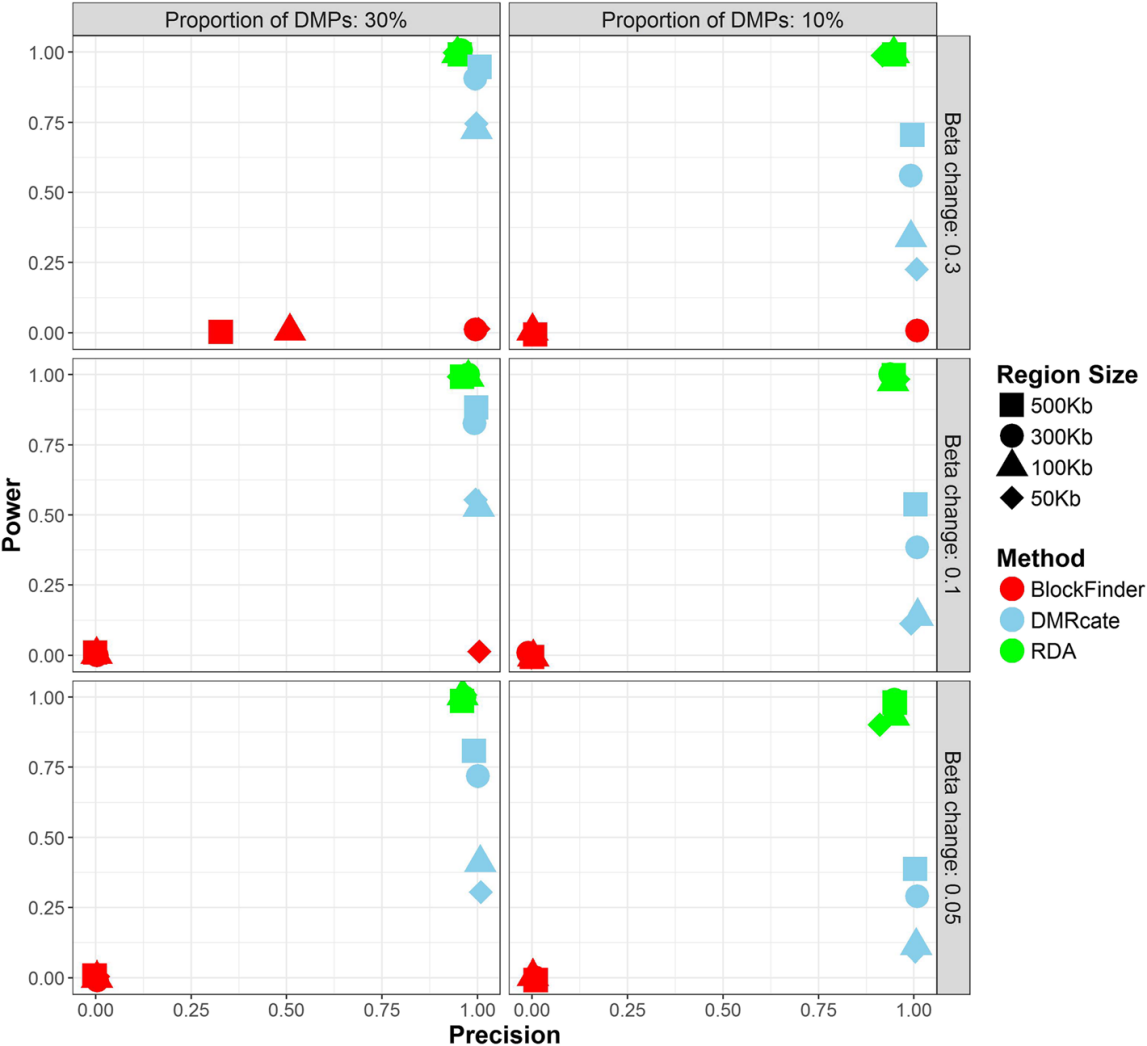
Precision and power of RDA, DMRcate, and blockFinder for simulated sets of 40 samples. To be considered a true positive, DMRcate regions should comprise at least 50% of the simulated region. A DMRcate region including CpGs outside the DMR was considered as a false positive. DMRcate parameters were set to preserve the default smoothing window while allowing for DMRs as big as our target DMRs. Each sub figure represents a different scenario and each shape a different size of the simulated DMR. Results were derived from 200 simulations

DMRcate had high power for large effect sizes and regions and high precision in all scenarios (Fig. 1). blockFinder had very low power and precision in all scenarios (Fig. 1). RDA method showed the best results in all scenarios, showing a very high precision and power (Fig. 1). As expected, the R^2^ estimated with RDA method was related to the proportion of simulated DMPs and its beta change, but not with the region size (Table 1). When performing the analyses in random regions, we observed that RDA properly controls type I error (5% of false positive results) while DMRcate and blockFinder underestimated it (~0% false positive results) Similar conclusions can be obtained for sample sizes equal to 10 and 100 (Additional file 1: Figures S5, S8 and Tables S1-S2).

Overall, Bumphunter only performed well in detecting bumps when the effect size was large. While DMRcate can be used to detect large DMRs, we have to balance accuracy of DMR boundaries and power. We can prioritize power by configuring DMRcate using the extended window; as a result, DMRs will be larger than our target region. On the other hand, we can prioritize precision and use the standard window, in which case DMRcate will be unable to detect the entire target region as a DMR. Independently of the parameters, DMRcate showed the best performance when the proportion of DMPs was large. blockFinder showed very poor performance in all simulations. Finally, RDA showed good performance in all situations and conditions.

### Real data analysis: BRCA dataset

We used the BRCA (breast cancer) data from TCGA (http://cancergenome.nih.gov/) to assess the methods’ performance. We used TCGAbiolinks to obtain data for 466 samples with Illumina 450 K methylation and clinical data [27]. We studied the methylation pattern of the human epidermal growth factor receptor 2 (HER2) region (chr17:37,700,000–38,000,000). Our aim was to establish whether HER2 status (positive and negative) has differentially methylated our region of interest. Besides the DMR methods, we also run a single probe analysis to get an estimate of the number of differentially methylated probes. Bumphunter and blockFinder were run with 1000 permutations and setting the minimum effect size to consider a probe in a bump/block to 0.1. DMRcate was run using smoothing and RDA was run using M-values and with 10,000 permutations to compute *p*-values.

**Fig. 2:**
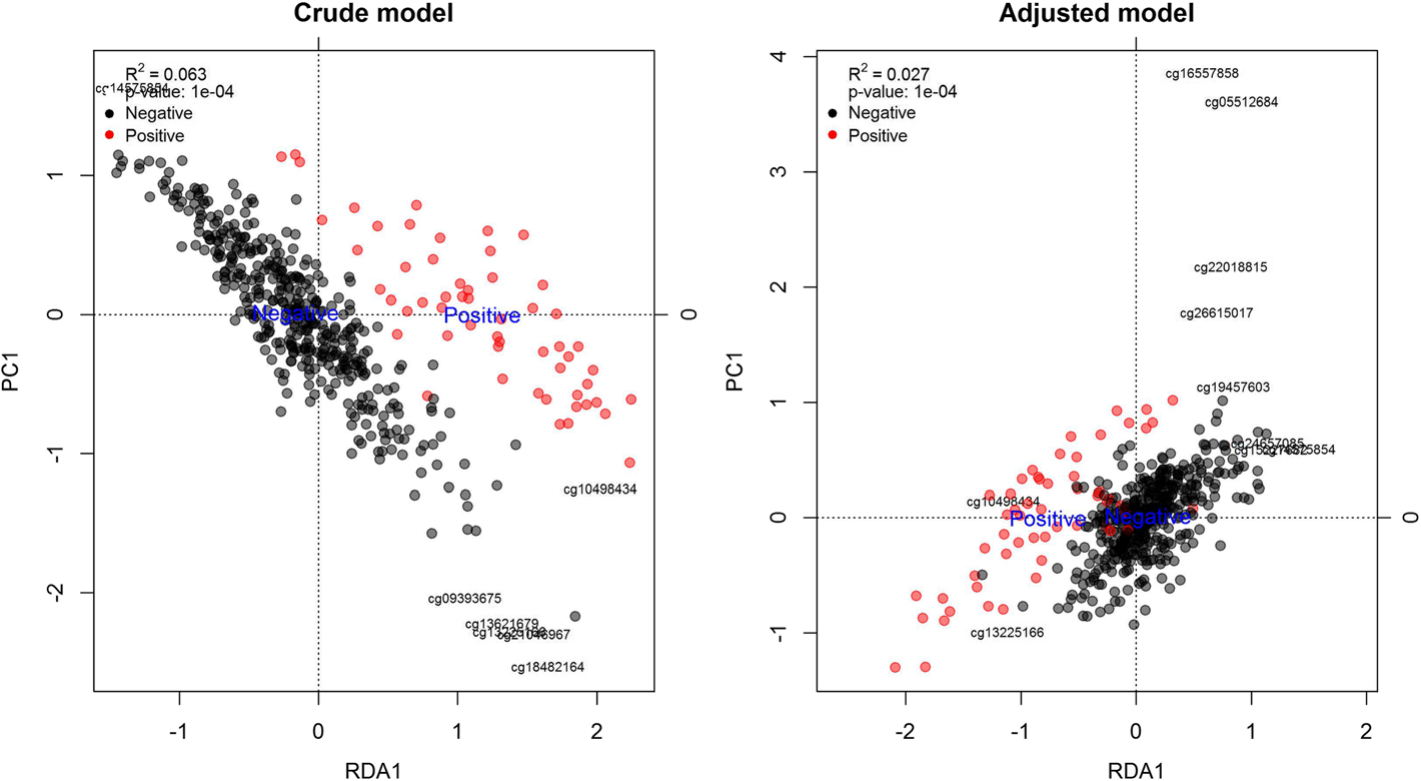
RDA biplot of the HER2 region (BRCA dataset) for crude and adjusted models. The adjusted model also includes surrogate variables in the analysis. The points represent the samples and are coloured according to HER2 status (HER2-, Negative; HER2+, Positive). Negative and Positive labels are placed at the centroid of their respective groups. CpGs most strongly associated with the RDA components are represented with labels. R^2^ is the proportion of variance in methylation data explained by the model. The *p*-value of the RDA model was computed by sampling the HER2 sample status variable

We first run the different methods using a crude model that only included HER2 status. Single probe analysis of methylation data detected 19,083 DMPs (FDR < 0.05) throughout the genome. Our region of interest contained 147 DMPs (~67% of CpGs included in the HER2 region). Interestingly, the CpGs with the lowest *p*-values were found in the HER2 region (Additional file 1: Figure S11). Bumphunter detected 359 significant bumps (FDR < 0.05) in the genome, of which 14 were in our region of interest. These bumps contained 40 CpGs or ~18% of the total CpGs. blockFinder found 35 significant blocks (FDR < 0.05), two of them in chromosome 17. One block comprised 30Kb and the other just one CpG. DMRcate returned 1414 DMRs ranging from 7 bases to 2.4 Mb, but mostly with a width of few Kb or a width around a Mb (Additional file 1: Figure S12). The top DMR detected by DMRcate (based on the Stouffer *p*-value) was 2.4 Mb long and included our target region. RDA analysis of the HER2 region showed a significant R^2^ (0.063, *p*-value: < 10^−4^). HER2+ and HER2- samples were clearly separated in the RDA representation (Fig. 2). The probability of finding a region of the same size and a higher R^2^ was <10^−4^, indicating that there is a very low probability of observing a region of this size that explains more than 5.8% of the variability in methylation.

**Fig. 3:**
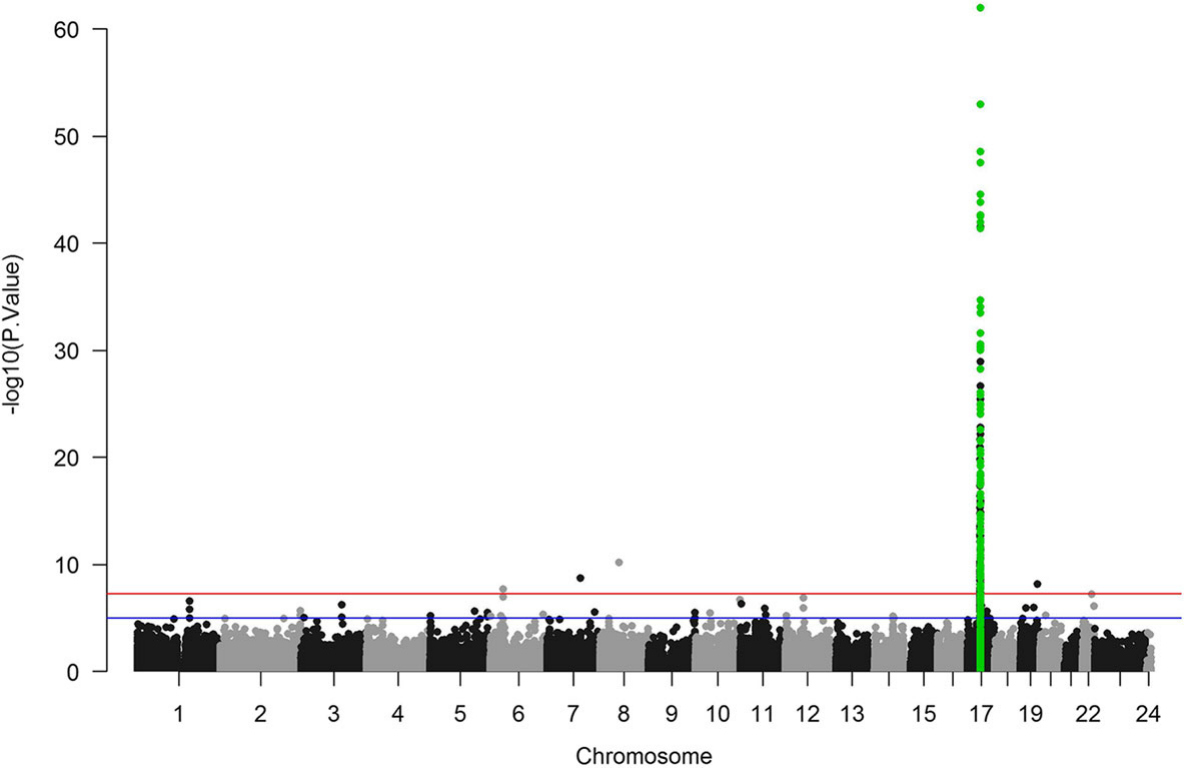
Manhattan plot of the adjusted analysis in the BRCA dataset analysis. These results reflect the statistical significance of the association between each CpG and HER2 status. CpGs are ordered by chromosome and position. CpGs in the HER2 region are highlighted in green

We also run all the methods using an adjusted model that contained HER2 and 53 surrogate variables obtained with smartSVA package. Single probe analysis returned considerably fewer DMPs (450) throughout the genome. Our region of interest contained 116 DMPs, representing 53% of the region CpGs. The CpGs with the most significant *p*-values were also found in the HER2 region (Fig. 3). Bumphunter detected 12 significant bumps, of which 9 were in our target region. These bumps comprised 33 CpGs or around 15% of the CpGs in this region. blockFinder detected three significant blocks, all of them in our target region comprising 32Kb. DMRcate called 7 regions as DMRs, most of which were shorter than 1 kilobase. DMRcate’s top DMR was 1.7 Mb in length and contained our target region. RDA analysis returned a significant but smaller R^2^ (0.027, *p*-value: < 10^−4^). The probability of finding a similar-sized region with higher R^2^ was also very low (<10^−4^). The RDA plot also separated the samples by HER2 subtype, but less clearly than using the crude model (Fig. 3). This may indicate that some of the observed differences can be explained by unobserved or technical variables.

**Fig. 4:**
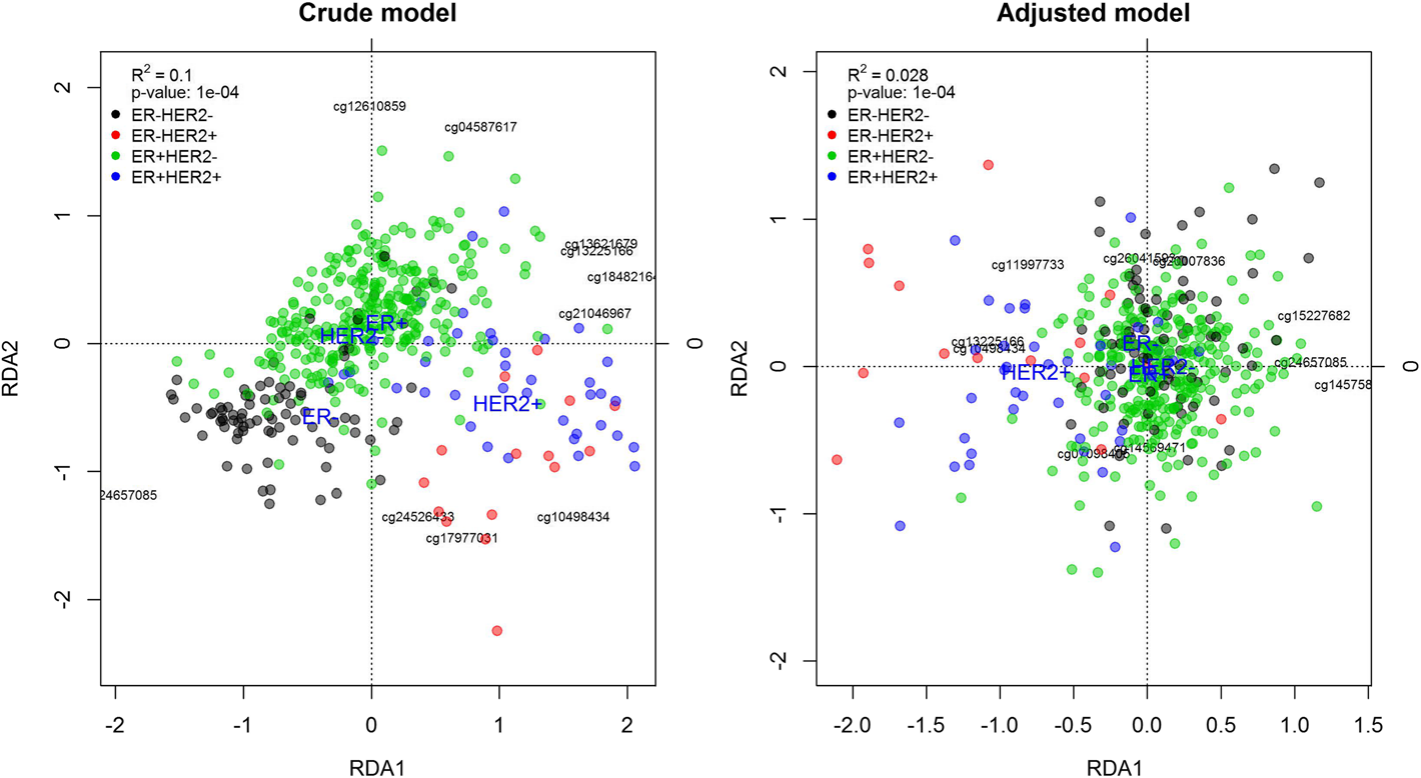
RDA biplot of the HER2 region (BRCA dataset) using HER2 and ER sample status. The adjusted model also includes surrogate variables in the analysis. The points represent the samples and are coloured according to the combination of HER2 and ER status. ER-, ER+, HER- and HER+ labels are placed at the centroid of their respective groups. The CpGs most associated with the RDA components are represented with labels. R^2^ is the proportion of variance in methylation data explained by the model. The *p*-value of the RDA model was computed by sampling the HER2 and ER sample status variables

RDA analysis including HER2 and ER subtype returned a higher and statistically significant R^2^ (0.100, *p*-value: < 10^−4^). It was also very unlikely to find regions of this size with the same or higher R^2^ (*p*-value: < 10^−4^). In the RDA representation, the samples were separated by HER2 subtype (Fig. 4). Separation by ER sample subtype was more evident in HER- than HER+ samples. When we included surrogate variables in the model, the R^2^ was reduced to the value of HER2 only model (0.028, *p*-value: < 10^−4^). However, the probability of finding a region with a higher R^2^ was still very low (<10^−4^). The RDA plot clearly separated samples by HER2 sample subtype in the first RDA component (Fig. 4). Only HER2+ samples were separated by ER sample subtype in the second component.

## Discussion

We have tested the performance of Bumphunter, blockFinder, DMRcate and our new method to detect large DMRs in six simulated scenarios and in a real cancer dataset.

Bumphunter failed to detect that the target region was a DMR in both datasets. Due to its implementation, Bumphunter can only detect small DMRs (around 1Kb), which makes it unsuitable for calling large DMRs. In our simulation, it failed to detect small methylation changes and only included all DMPs in bumps when the effect size was large (0.3). This poor performance may be because we introduced the DMPs in random positions in our target region. When the effect size was large, the individual signal of each DMP was enough to call a bump, but when the methylation change was low, Bumphunter could not distinguish the signal from the background noise. One of Bumphunter’s strength is the inclusion of a method to test bumps’ significance. Although computing these *p*-values is very time and memory consuming, it provides a clear criterion for differentiating relevant from spurious DMRs.

blockFinder, Bumphunter’s adaptation for large regions, detected very few blocks in all situations, mainly because blockFinder only uses open sea probes. In our simulation, the DMPs were randomly distributed, so if DMPs were not open sea probes, blockFinder was unable to detect a DMR. The same problem applied to the real dataset, where blockFinder only detected a significant DMR in the crude model.

DMRcate showed good performance in both datasets. In the simulations, DMRcate showed better results when the signal was strong (high proportion of DMPs, high methylation changes, and very large regions), and we suggest that this is due to the smoothing process. Where there was a low proportion of DMPs or their methylation change was small, the smoothing removes this signal. The smoothing also explains why DMRcate performed better in the largest regions, even though it is designed to detect small DMRs. DMRcate is designed to detect groups of differentially methylated probes that are near each other. In this situation, DMRcate can detect weak signals because the smoothing compensates probes with small changes with probes with greater changes. However, the DMPs in our simulation were randomly distributed throughout the region and thus were unlikely to be grouped. In this situation, the smoothing weakens the individual signal of each probe by combining the signal of the DMPs with general noise. In large regions, the total number of DMPs is higher, and the region can be detected even after smoothing. The results from our real dataset differed between the crude and adjusted model, in that there were many differentially methylated CpGs in the crude model. Since we changed the clustering parameter to allow us to group probes as far away as the total length of the HER2 region, DMRcate detected very large DMRs. In the adjusted model, there were very few DMPs and all the DMRs were small (around 1Kb) except for our target region. In both cases, DMRcate called a DMR including our target region, although the called region was much larger. Again, the smoothing process is responsible for this behaviour: the marked methylation changes in our target region compensate for the noise of nearby probes, leading to detection of larger DMR than our target region. As a result, there is a greater risk of false positives. Where our target region is close to a region with marked methylation changes, DMRcate can include the target region within the DMR, even if the target region does not have methylation changes. Another issue is that DMRcate does not estimate the significance of the DMR or the magnitude of the effect, and the lack of a *p*-value makes it difficult to distinguish spurious from true associations.

Our RDA method showed good performance in the simulations, with very high power and precision in all scenarios, even with small sample sizes (10 samples). Our method provides an overall estimate of the association between the methylation and the factor of interest: the R^2^. We found that this estimate depends on the strength of the association between the methylation data and the factor, independent of sample size or region length. Consequently, the R^2^ estimate is useful for comparing results from different regions, and even for different experiments. In the simulations, the R^2^ ranged from 0.8 for the biggest association to 0.05 for the smallest. The R^2^ of the scenarios with very little association is very similar to what has been reported in the literature. For instance, in a work that studied the influence of genotype and environment on DNA methylation, they found that some important phenotypic variables (i.e. sex, age, blood cell counts, principal components of genotypes and some technical variables) together explained 17% of the total variance of the global DNA methylation.

In the real dataset, RDA was the only method that returned specific results for the target region. We got R^2^s of the same magnitude than the R^2^s of the small association scenarios, suggesting that the association of the methylation with the cancer subtype was small but relevant. In addition, our method computes the probability of finding a similar region with a bigger R^2^, which is useful for assessing whether the association between the regional methylation pattern and our factor is specific to our region of interest. Another advantage is that our method can evaluate an entire linear model, allowing us to test the effects on methylation patterns of categorical variables with more than two levels, or the effects of different variables at the same time. The plotting function we have included in the MEAL package allows the user to obtain a quick overview of how different categorical variables model the methylation pattern of the region.

We must notice that RDA assumptions must be checked before running the analysis. As stated in the Implementation section, RDA requires normality in the outcome variable (e.g. methylation data) and linearity between methylation and factor variable. In order to hold normality assumption, we analyze M-values instead of beta values (*runRDA* function by default transforms beta values into M-values by using logit2 transformation). Linearity can be checked by visual inspection. In our particular example (TCGA methylation data) linearity is satisfied since we are analyzing a factor variable with two categories.

In studies to date, the association between variables and methylation in large regions has been assessed using DMPs or small DMRs [17, 18, 20]. In MEAL, we propose a method that returns an overall estimate of this association. One application could be to evaluate the effect of structural variants on methylation, and thereby it may be possible to propose mechanisms linking structural variants to diseases via regional changes instead of single changes. If we do not have a region of interest a priori, we can use RDA to complement current tools for detecting DMPs and small DMRs. This would involve performing a single probe analysis first, and, if we find a region with many DMPs or small DMRs, check this with RDA.

Our RDA method has also been successfully applied to expression data (Additional file 2), showing that regional analysis using RDA could be extended to other omics datasets.

## Conclusions

We propose RDA as a new tool for evaluating the effect of a variable on methylation in a large genomic region. Using simulated and real datasets, we show that our method performs better than the best state-of-the-art methods for detect DMRs. RDA returns an estimate of the magnitude of the regional association that is independent of sample size and region length, allowing the comparison of results obtained from different experiments. It also returns an estimate of the statistical significance of the association, which allows discarding spurious associations. In addition, our method can evaluate factors with more than two levels or the simultaneous effect of more than one continuous variable, which is not possible with the state-of-the-art methods. Finally, the application of the RDA method to gene expression, suggest that it could be applied to any other omics source, such as SNPs.

## Availability and requirements

**Project name:** MEAL.

**Project home page**: http://bioconductor.org/packages/release/bioc/html/MEAL.html

**Operating system(s):** Platform independent.

**Programming language:** R.

**Other requirements:** R 3.3.0 or higher.

**License:** Artistic-2.0.

**Any restrictions to use by non-academics:** none.

## Additional files

Additional file 1: Supplementary figures and tables. (PDF 1663 kb)

Additional file 2: RDA analysis applied to expression data. (PDF 1448 kb)

## Abbreviations

DMP: Differentially Methylated Probe
DMR: Differentially Methylated Region
ER: Estrogen Receptor
R^2^: R-squared
RDA: redundancy analysis
SNP: single nucleotide polymorphisms
SVA: Surrogate Variable Analysis
