# Supplementary material for: Redundancy analysis allows improved detection of methylation changes in large genomic regions
Source: BMC Bioinformatics. 2017 Dec 14;18:553. doi: 10.1186/s12859-017-1986-0 (PMC5729265; doi:10.1186/s12859-017-1986-0)
Supplement: Supplementary file 1 — Supplementary figures and tables. (PDF 1663 kb) [file 12859_2017_1986_MOESM1_ESM.pdf]

Additional File 1: Supplementary figures and tables

Outline

Figure S1 2

Figure S2 3

Figure S3 4

Figure S4 5

Figure S5 6

Figure S6 7

Figure S7 8

Figure S8 9

Figure S9 10

Figure S10 11

Figure S11 12

Figure S12 13

Table S1 14

Table S2 15

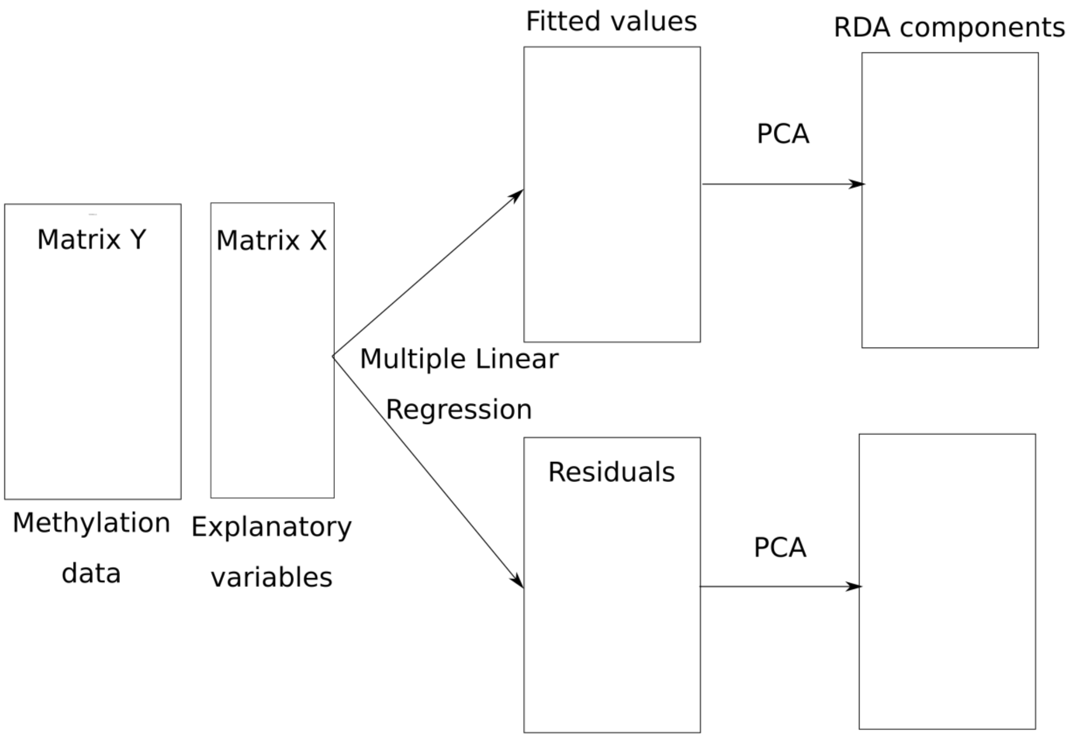

Figure S1**: Redundancy Analysis algorithm.** For each variable of matrix Y, a multiple linear regression is run including all variables of matrix X. As a result, two matrices are generated, one with the fitted values and another with the residuals. A Principal Component Analysis is run to both matrices.

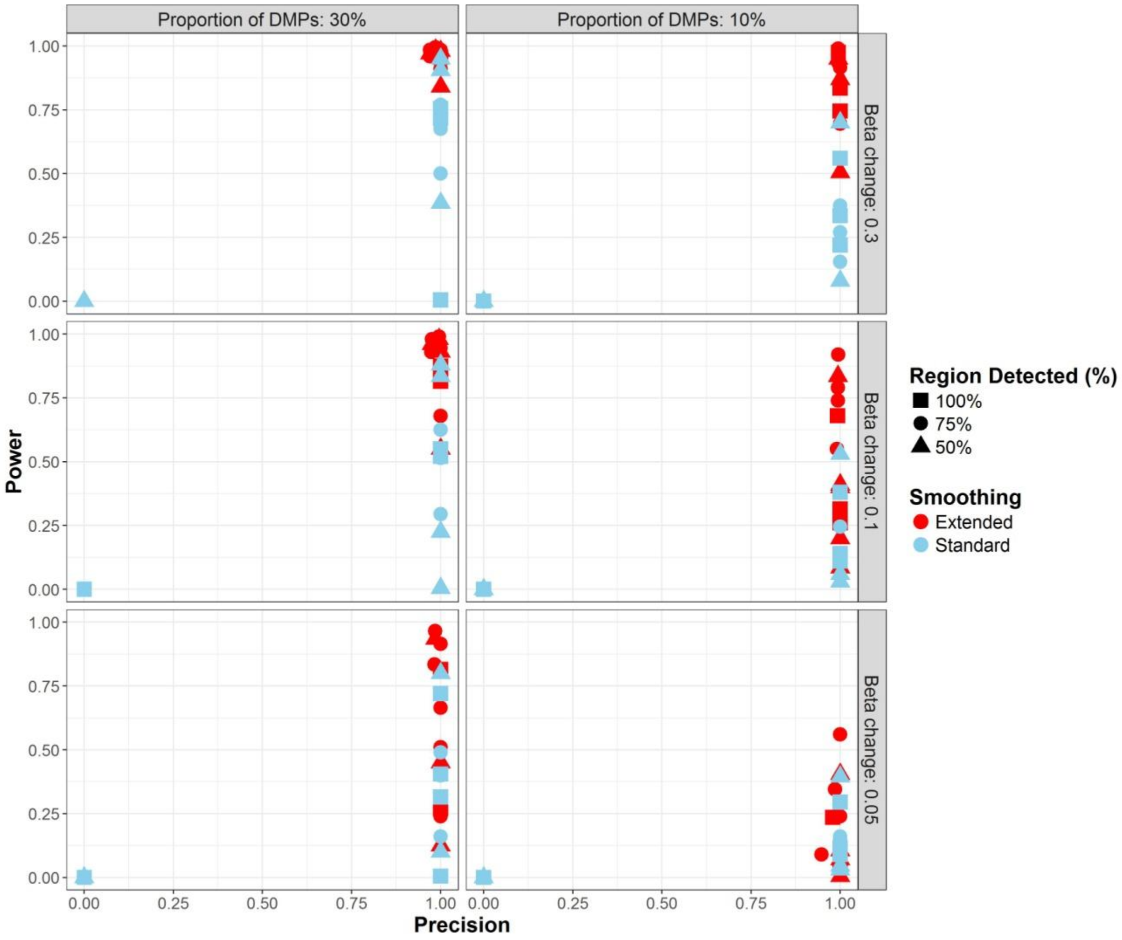

Figure S2**: DMRcate's precision and recall using extended and standard smoothing (sets of 40 samples).** DMRcate parameters in standard smoothing were set to preserve the default smoothing window but allowing DMRs as big as our target DMRs. DMRcate parameters in extended smoothing increased the smoothing window accordingly to the target DMR size. Each shape is a different threshold to consider a DMRcate region as a true positive (DMRcate region size is at least 50%, 75% or 100% of the size of the simulated DMR). A DMRcate region including CpGs outside the simulated region was considered as a false positive. Each subgraph represents a different scenario. Results were computing from 200 simulations.

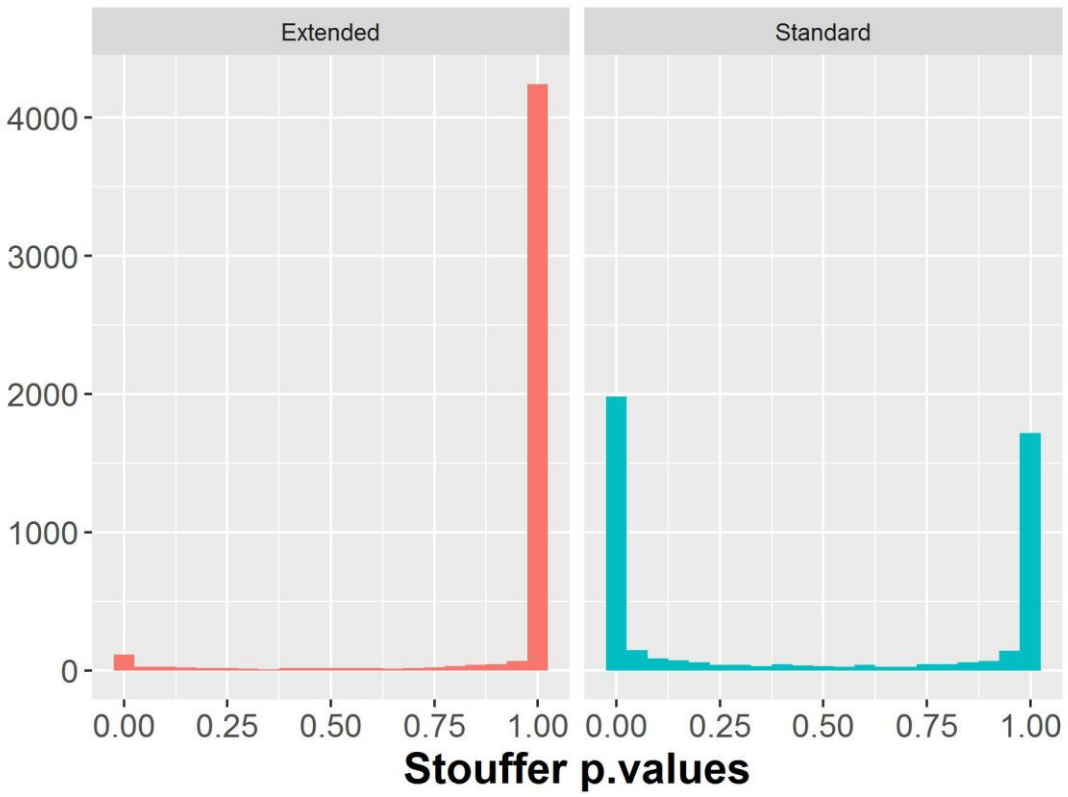

Figure S3**: Distribution Stouffer-p.values using extended and standard smoothing (sets of 40 samples).** We only included DMRs overlapping with the target region. The distribution includes the results of the six different scenarios.

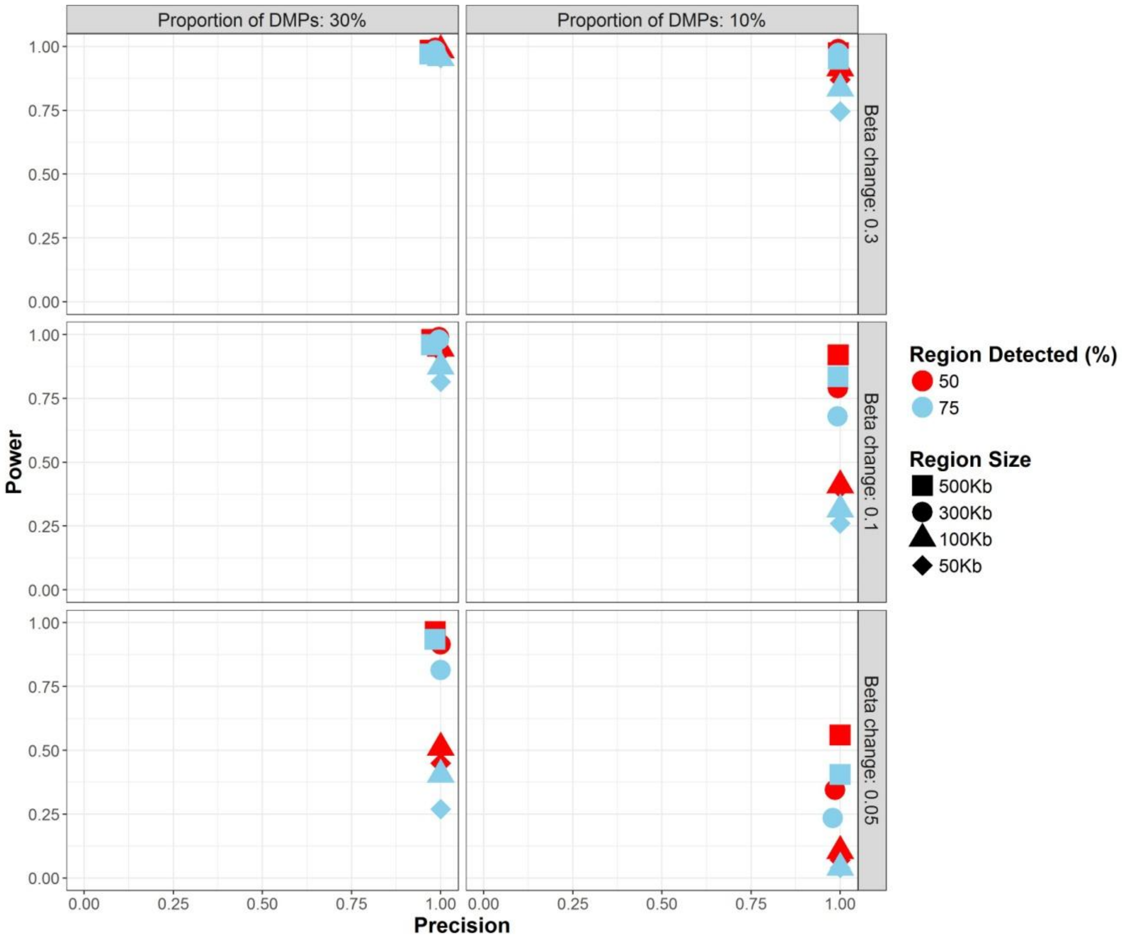

Figure S4: **Precision and recall DMRcate using different detection thresholds (sets of 40 samples).** The detection threshold is the minimum size that a DMRcate region should have to be considered a true positive (50% or 75% of the simulated DMR size). A DMRcate region including CpGs outside the DMR was considered as a false positive. DMRcate parameters were set to preserve the default smoothing window but allowing DMRs as big as our target DMRs. Each subgraph represents a different scenario and each shape a different size of the simulated DMR. Results were computing from 200 simulations.

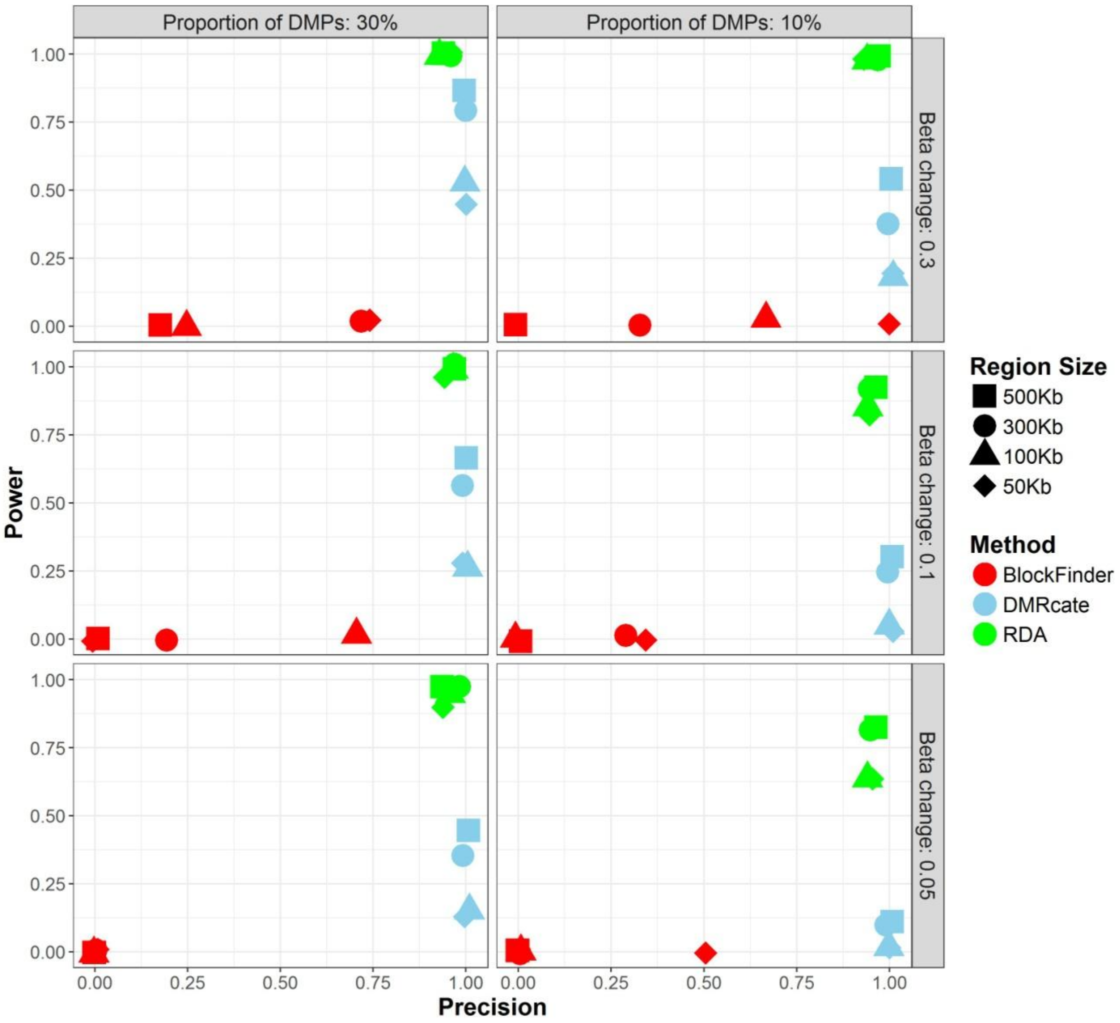

Figure S5**: Precision and recall of RDA, DMRcate and blockFinder for simulated sets of 10 samples.** DMRcate regions and blockFinder blocks should comprise at least 50% of the simulated region to be considered a true positive. A DMRcate region and a blockFinder block including CpGs outside the DMR was considered as a false positive. DMRcate parameters were set to preserve the default smoothing window but allowing DMRs as big as our target DMRs. Each subgraph represents a different scenario and each shape a different size of the simulated DMR. Results were computing from 200 simulations.

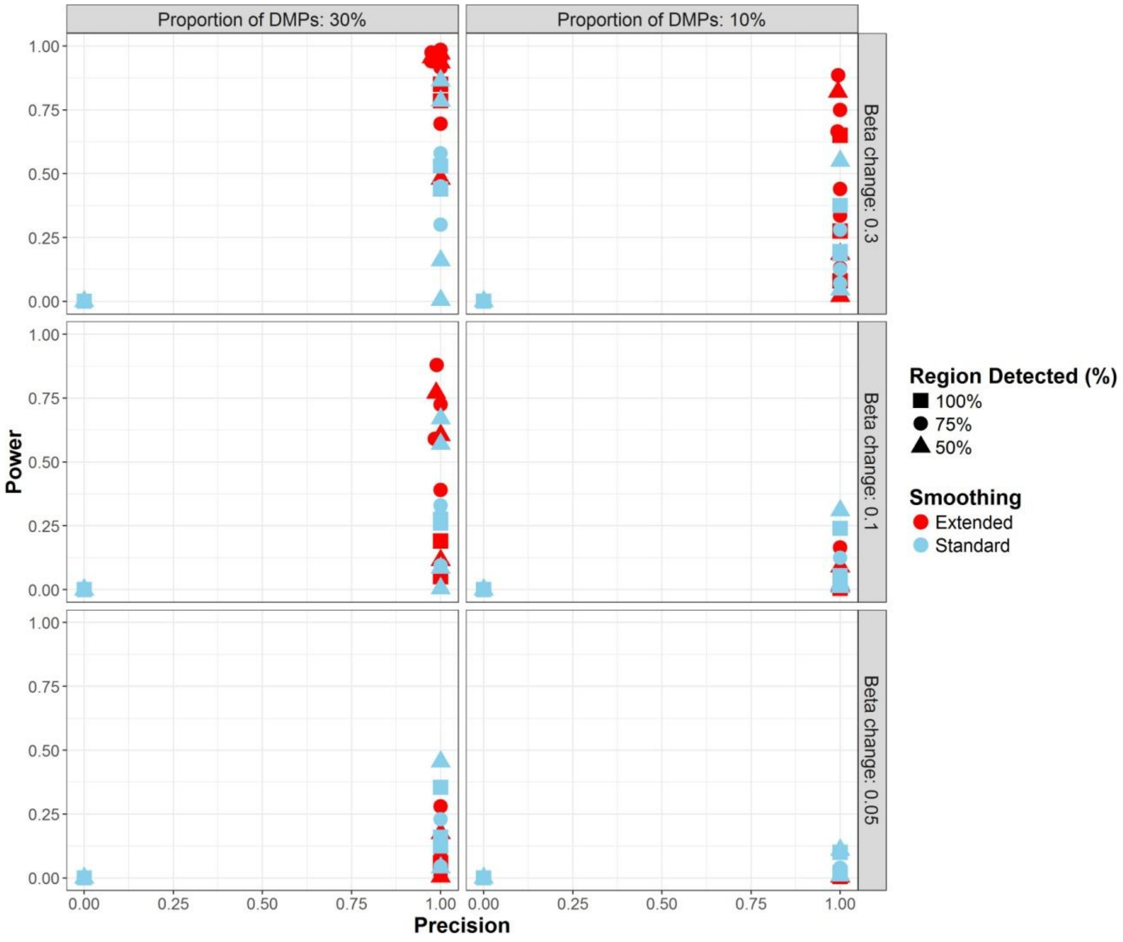

Figure S6**: DMRcate's Precision and recall using extended and standard smoothing (sets of 10 samples).** DMRcate parameters in standard smoothing were set to preserve the default smoothing window but allowing DMRs as big as our target DMRs. DMRcate parameters in extended smoothing increased the smoothing window accordingly to the target DMR size. Each shape is a different threshold to consider a DMRcate region as a true positive (DMRcate region size is at least 50%, 75% or 100% of the size of the simulated DMR). A DMRcate region including CpGs outside the simulated region was considered as a false positive. Each subgraph represents a different scenario. Results were computing from 200 simulations.

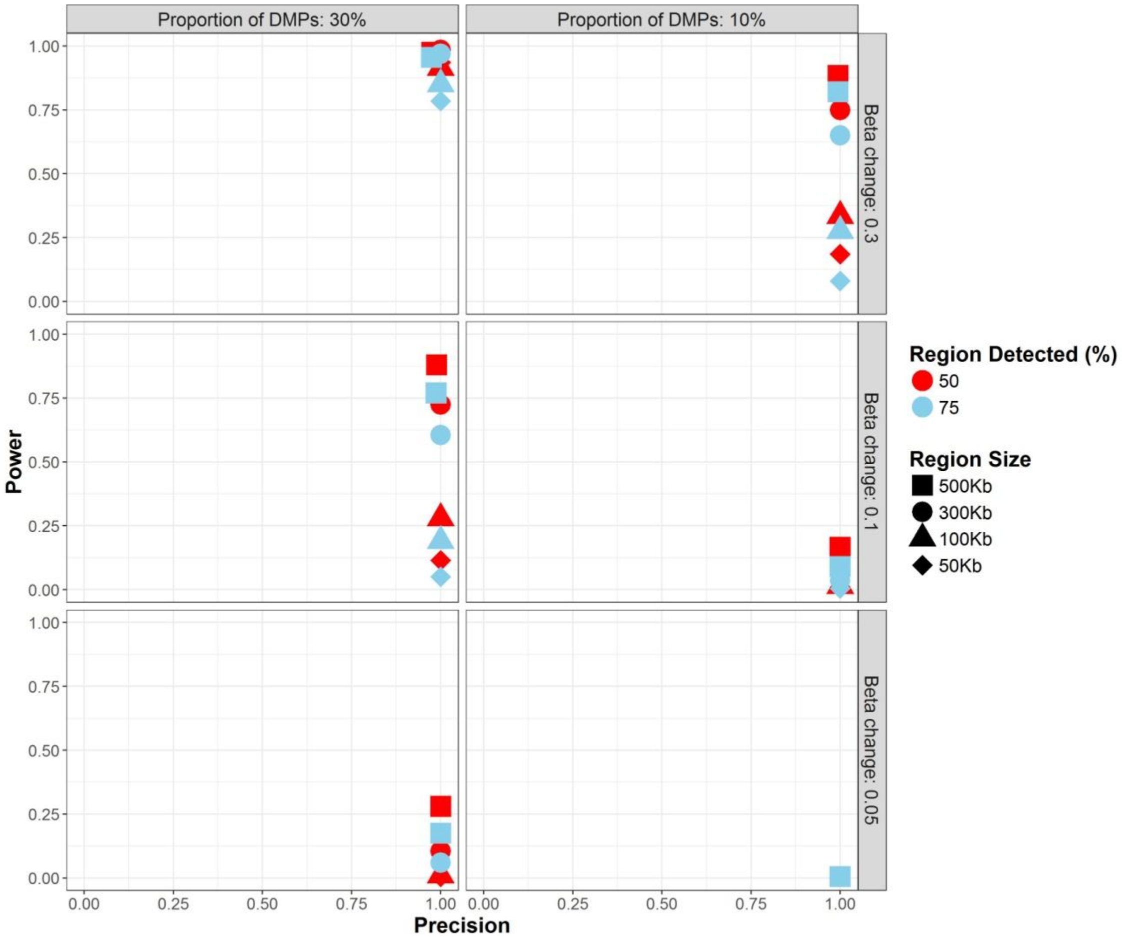

Figure S7**: DMRcate's precision and recall using different detection thresholds (sets of 10 samples).** The detection threshold is the minimum size that a DMRcate region should have to be considered a true positive (50% or 75% of the simulated DMR size). A DMRcate region including CpGs outside the DMR was considered as a false positive. DMRcate parameters were set to preserve the default smoothing window but allowing DMRs as big as our target DMRs. Each subgraph represents a different scenario and each shape a different size of the simulated DMR. Results were computing from 200 simulations.

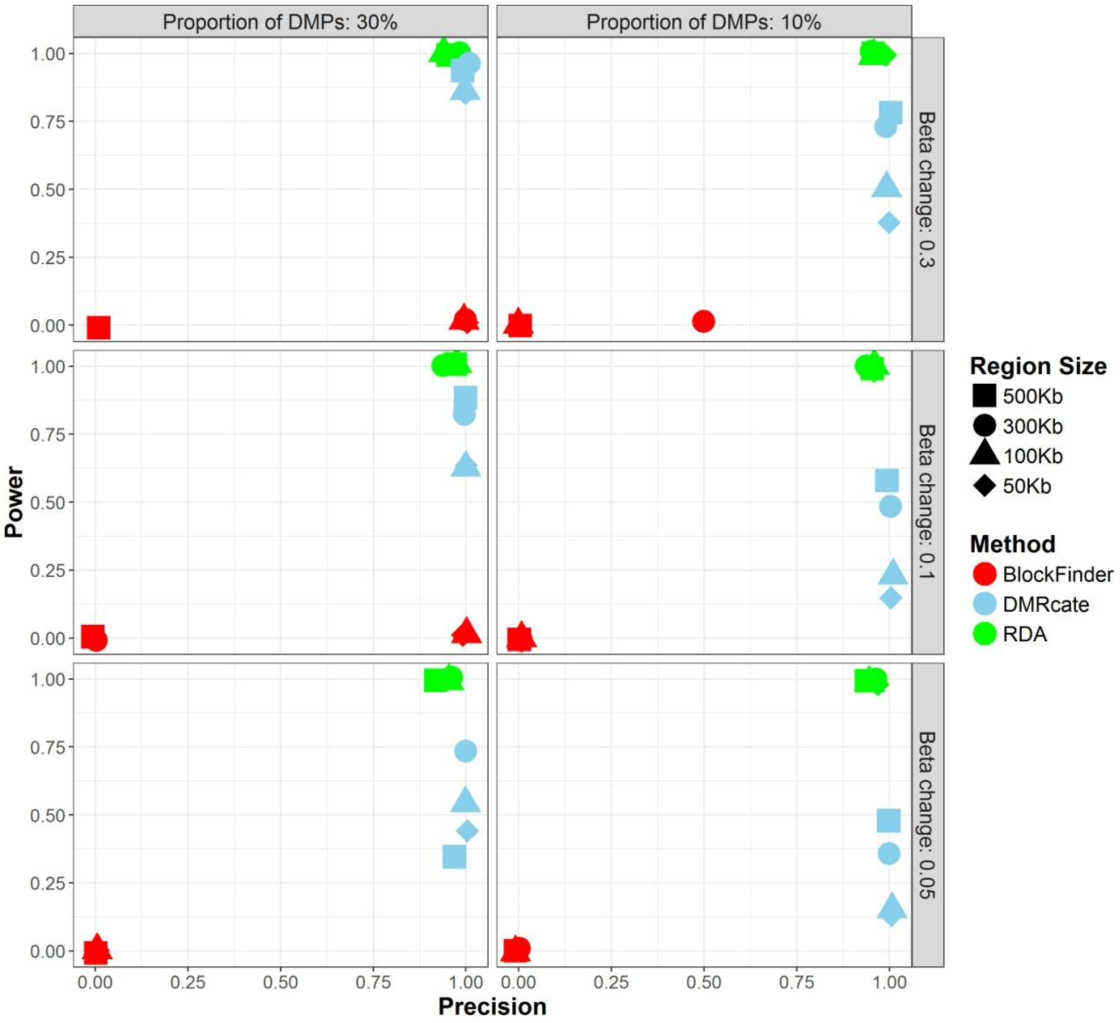

Figure S8: **Precision and recall of RDA, DMRcate and blockFinder for simulated sets of 100 samples.** DMRcate regions and blockFinder blocks should comprise at least 50% of the simulated region to be considered a true positive. A DMRcate region and a blockFinder block including CpGs outside the DMR was considered as a false positive. DMRcate parameters were set to preserve the default smoothing window but allowing DMRs as big as our target DMRs. Each subgraph represents a different scenario and each shape a different size of the simulated DMR. Results were computing from 200 simulations.

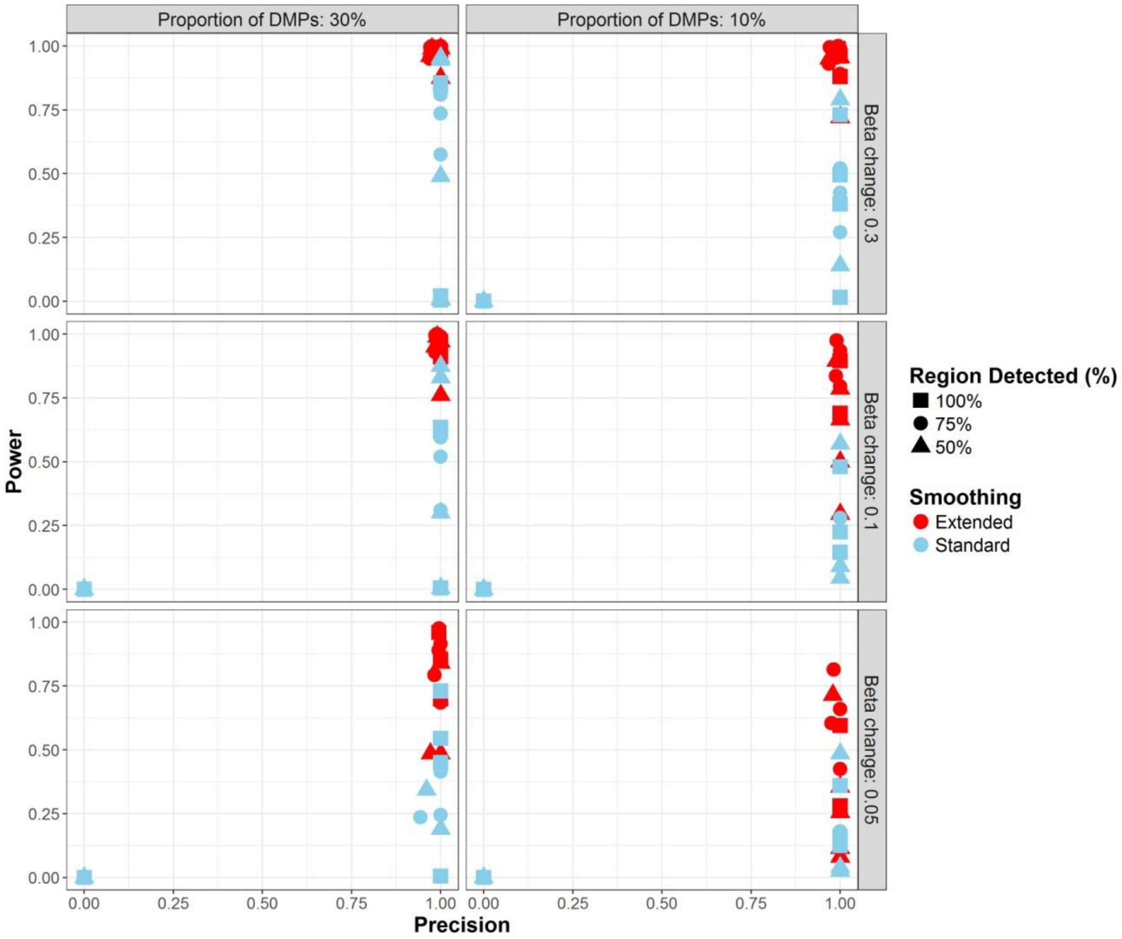

Figure S9**: DMRcate's precision and recall using extended and standard smoothing (sets of 100 samples).** DMRcate parameters in standard smoothing were set to preserve the default smoothing window but allowing DMRs as big as our target DMRs. DMRcate parameters in extended smoothing increased the smoothing window accordingly to the target DMR size. Each shape is a different threshold to consider a DMRcate region as a true positive (DMRcate region size is at least 50%, 75% or 100% of the size of the simulated DMR). A DMRcate region including CpGs outside the simulated region was considered as a false positive. Each subgraph represents a different scenario. Results were computing from 200 simulations.

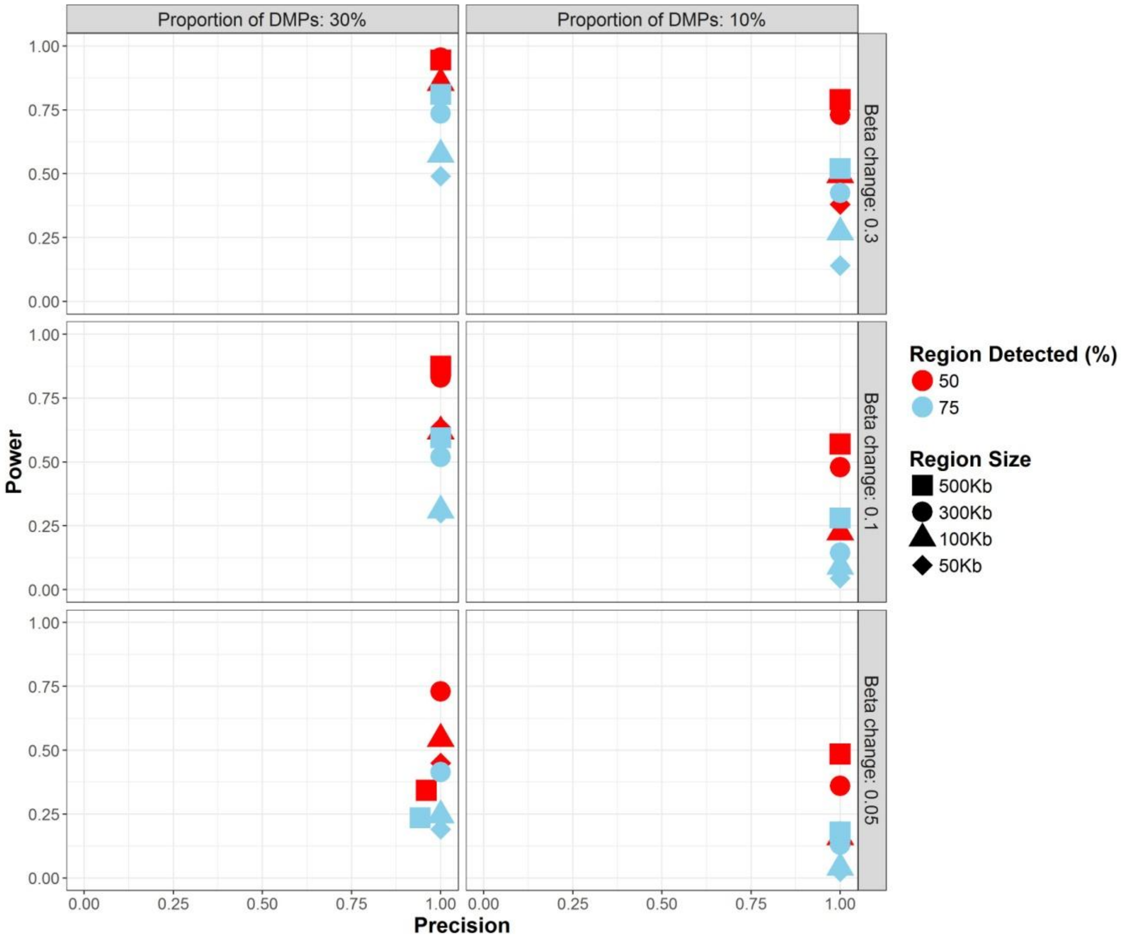

Figure S10**: DMRcate's Precision and recall using different detection thresholds (sets of 100 samples sets).** The detection threshold is the minimum size that a DMRcate region should have to be considered a true positive (50% or 75% of the simulated DMR size). A DMRcate region including CpGs outside the DMR was considered as a false positive. DMRcate parameters were set to preserve the default smoothing window but allowing DMRs as big as our target DMRs. Each subgraph represents a different scenario and each shape a different size of the simulated DMR. Results were computing from 200 simulations.

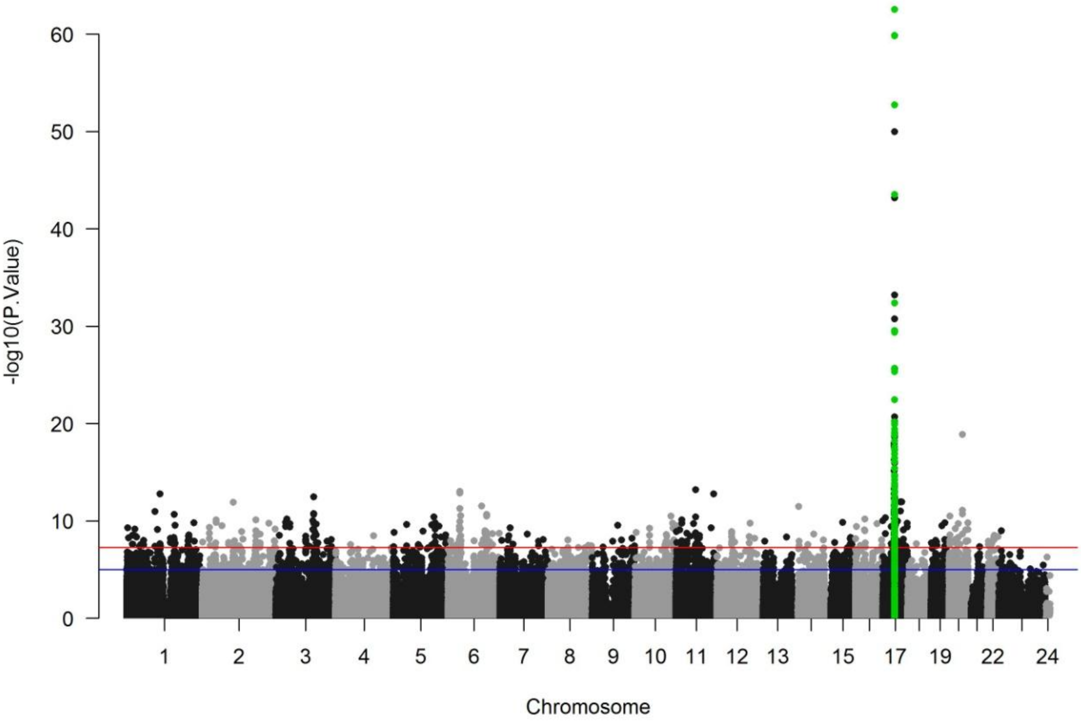

Figure S11**: Manhattan plot of the crude analysis in the BRCA dataset analysis.** These results refer to the statistical significance of the association between each CpG and the HER2 status. CpGs are ordered by chromosome and position. CpGs belonging to the HER2 region are highlighted in green.

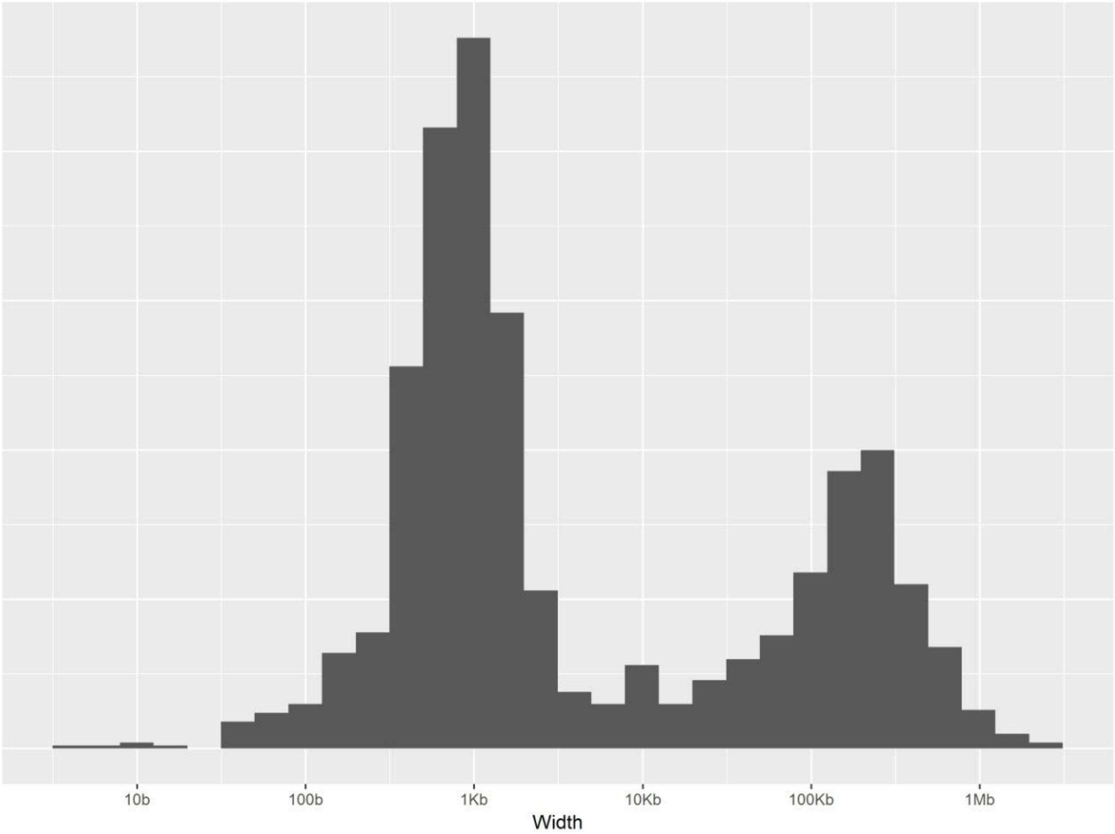

Figure S12**: Width distribution of DMRcate’s DMRs using crude model.** We included all the DMRs detected by DMRcate in the crude model. The x axis is the log10 of the width.

Table S1: **Methylation region analysis using current methods (10 samples)**

Region size	Sim. DMP %	Beta change	CpGs in Bumps (%)	R^2 (sd)	
				Target region	Random region
**500Kb**	30	0.3	4.94	0.705 (0.108)	0.110 (0.034)
	30	0.1	0.67	0.506 (0.106)	0.110 (0.029)
	30	0.05	0.03	0.417 (0.141)	0.114 (0.041)
	10	0.3	0.52	0.518 (0.087)	0.106 (0.028)
	10	0.1	0.03	0.348 (0.099)	0.108 (0.035)
	10	0.05	0.01	0.278 (0.145)	0.113 (0.033)
**300Kb**	30	0.3	5.23	0.726 (0.102)	0.109 (0.039)
	30	0.1	0.74	0.492 (0.107)	0.113 (0.042)
	30	0.05	0.01	0.416 (0.134)	0.107 (0.032)
	10	0.3	0.59	0.502 (0.100)	0.113 (0.040)
	10	0.1	0.09	0.337 (0.111)	0.109 (0.038)
	10	0.05	0.00	0.276 (0.142)	0.109 (0.037)
**100Kb**	30	0.3	4.78	0.731 (0.120)	0.123 (0.065)
	30	0.1	0.64	0.506 (0.114)	0.111 (0.041)
	30	0.05	0.00	0.395 (0.114)	0.109 (0.049)
	10	0.3	0.52	0.531 (0.109)	0.107 (0.046)
	10	0.1	0.02	0.327 (0.113)	0.109 (0.043)
	10	0.05	0.04	0.238 (0.108)	0.107 (0.043)
**50Kb**	30	0.3	5.28	0.740 (0.111)	0.117 (0.048)
	30	0.1	0.23	0.514 (0.123)	0.109 (0.047)
	30	0.05	0.00	0.387 (0.129)	0.104 (0.043)
	10	0.3	0.43	0.527 (0.119)	0.112 (0.052)
	10	0.1	0.00	0.325 (0.124)	0.110 (0.043)
	10	0.05	0.00	0.239 (0.108)	0.108 (0.043)

Values are the mean of the 200 simulations. Sim. DMP %: percentage of DMPs introduced in the simulation. Beta change: DMP's beta change. CpGs in Bumps: (%): proportion of CpGs of the modified region that are inside a bump with a fwer < 0.05. R^2: R^2 estimate of RDA model. Target region: region that includes our simulated DMPs. Random region: region without any of the simulated DMPs.

Table S2: **Methylation region analysis using current methods (100 samples)**

Region size	Sim. DMP %	Beta change	CpGs in Bumps (%)	R^2 (sd) Target region	R^2 (sd) Random region
**500Kb**	30	0.3	29.60	0.646 (0.131)	0.010 (0.004)
	30	0.1	4.99	0.418 (0.093)	0.010 (0.003)
	30	0.05	1.03	0.335 (0.134)	0.014 (0.031)
	10	0.3	9.65	0.439 (0.091)	0.010 (0.003)
	10	0.1	0.51	0.244 (0.080)	0.010 (0.004)
	10	0.05	0.07	0.174 (0.116)	0.010 (0.004)
**300Kb**	30	0.3	29.50	0.664 (0.123)	0.010 (0.003)
	30	0.1	5.02	0.401 (0.081)	0.010 (0.004)
	30	0.05	0.94	0.317 (0.109)	0.010 (0.004)
	10	0.3	9.54	0.438 (0.096)	0.010 (0.004)
	10	0.1	0.55	0.232 (0.087)	0.011 (0.004)
	10	0.05	0.14	0.159 (0.102)	0.010 (0.004)
**100Kb**	30	0.3	29.20	0.696 (0.109)	0.010 (0.005)
	30	0.1	4.41	0.414 (0.079)	0.010 (0.004)
	30	0.05	1.13	0.307 (0.098)	0.010 (0.005)
	10	0.3	9.13	0.431 (0.118)	0.011 (0.005)
	10	0.1	0.31	0.232 (0.102)	0.010 (0.004)
	10	0.05	0.10	0.140 (0.102)	0.010 (0.006)
**50Kb**	30	0.3	28.80	0.701 (0.116)	0.010 (0.005)
	30	0.1	4.72	0.406 (0.106)	0.010 (0.005)
	30	0.05	1.05	0.311 (0.136)	0.010 (0.005)
	10	0.3	8.98	0.422 (0.117)	0.009 (0.004)
	10	0.1	0.47	0.228 (0.110)	0.010 (0.005)
	10	0.05	0.08	0.130 (0.112)	0.010 (0.004)

Values are the mean of the 200 simulations. Sim. DMP %: percentage of DMPs introduced in the simulation. Beta change: DMP's beta change. CpGs in Bumps: (%): proportion of CpGs of the modified region that are inside a bump with a fwer < 0.05. R^2: R^2 estimate of RDA model. Target region: region that includes our simulated DMPs. Random region: region without any of the simulated DMPs.
