# Supplementary material for: Redundancy analysis allows improved detection of methylation changes in large genomic regions
Source: BMC Bioinformatics. 2017 Dec 14;18:553. doi: 10.1186/s12859-017-1986-0 (PMC5729265; doi:10.1186/s12859-017-1986-0)
Supplement: Supplementary file 2 — RDA analysis applied to expression data. (PDF 1448 kb) [file 12859_2017_1986_MOESM2_ESM.pdf]

Additional file 2: RDA analysis applied to expression data

Introduction

MEAL can also apply our RDA method to gene expression data. The goal of this section is to illustrate the performance of our RDA method with expression data in a simulated and a real dataset.

Results and discussion

Expression simulated data

Differential gene expression analysis detected almost all the simulated changes in the sets with 40 samples (Table S1). DE proportion was almost the same than the number of genes with a simulated change. Our method performed very well in all the scenarios (recall and precision bigger than 0.9). As it happened with methylation data, R^2 was affected by the effect size and proportion of DEG. R^2 in random regions was low and it was not affected by the scenario. With a sample size of 100, the results were very similar (Table S2). However, with the sample size of 10, recall was penalized in the most stringent scenario (Table S3). These results justify the extension of the RDA use to gene expression data.

TCGA

In this analysis, we studied the gene expression pattern of the HER2 region (chr17:37700000-38000000). Our aim was to establish whether HER2 status (positive and negative) changed the gene expression pattern in our region of interest.

Using the crude model, we found that 4064 were differentially expressed genes (Figure S1). Of those, eight were in our region of interest (all the region probes). RDA analysis returned a significant region R^2 (0.441, p-value: $< 10^{-5}$). The probability of finding a region with the same size and a bigger R^2 was really small ($< 10^{-5}$). HER2+ and

HER2- samples were clearly differentiated in RDA representation (

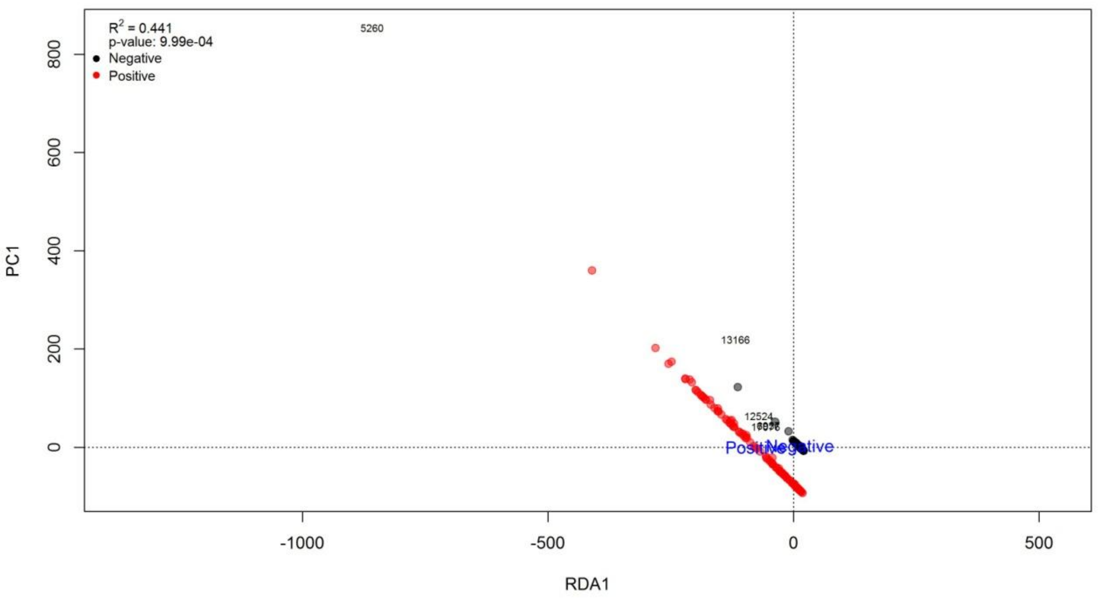

Figure S2). The adjusted model returned very similar results. We found that 4130 genes were differentially expressed and of those, five were in the HER2 region (62.5% of region genes, Figure S3). The RDA results were almost the same (R^2: 0.438, p-value < 10^{-5}, Figure S4). However, the probability of finding a region with the same size and a bigger R^2 was higher ($2 \cdot 10^{-3}$). These results suggest that the association between gene expression and cancer is stronger in the HER2 region than in the whole genome.

The inclusion of ER status in the model did not change the results. Using the crude model, RDA returned a big and significant R^2 (0.443, p-value < 10^{-5}). The probability of finding a region with the same size and a bigger R^2 was really small (< 10^{-5}). The adjusted model returned almost the same results (R^2: 0.443, p-value < 10^{-5}) and a higher probability of finding a region with the same size and a bigger R^2 was higher ($2 \cdot 10^{-3}$). In both cases, the samples

were only separated by HER2 status (Figure S5-

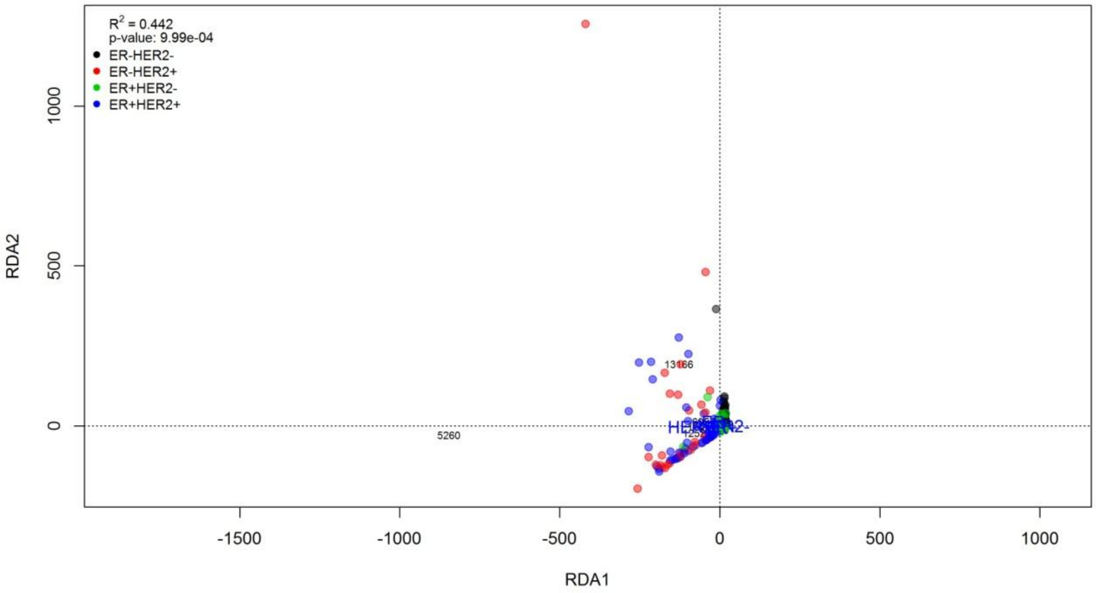

Figure S6). However, the groups were not as clear as in the model including only HER2 status.

Conclusions

In this document, we have evaluated the performance of our RDA method in gene expression data. The results are very similar to those obtained with methylation data, suggesting that our method can also be used in other omics data.

Methods

Simulation

In order to mimic real situations, expression values for expression probes were generated from a multinormal distribution with parameters estimated using real data obtained from GEO GSE53243 dataset (64 cell lines using Illumina HumanRef-8 v3.0 expression beadchip). We simulated expression values for all the expression probes of chromosome 19 and then we randomly generated differential expressed regions of 1Mb. To simulate the differential expressed regions, we introduced some expression probes differentially expressed depending on two groups (1 and 2), having the same sample size (5, 20 and 50). We simulated different scenarios based on three different effect size (i.e. log fold change: 1 and 0.5) and two different

percentage of DE genes (30% and 10%). Method's performance was evaluated in 200 simulated datasets.

Analysis

RDA was run using functions implemented in `MEAL`. The model used only included sample group. We run RDA with 1000 permutations to determine the model p-value and the region was differentially expressed region when p-value < 0.05.

RDA was performed in the simulated DMRs and in randomly chosen regions in order to assess whether type I error was controlled.

BRCA dataset

BRCA (breast cancer) data from TCGA (http://cancergenome.nih.gov/) was used to assess methods' performance. We downloaded 752 samples having Illumina HiSeq gene expression and HER2 status using `TCGAbiolinks` [1]. We studied the gene expression pattern of the human epidermal growth factor receptor 2 (HER2) region (chr17:37700000-38000000).

We first evaluated the effect of HER2 samples subtype (positive vs negative) on the regional gene expression pattern. Single probe methylation analysis and our proposed method were run using `MEAL`. We run two models: crude and adjusted. In the crude model, we only included HER2 sample subtype as covariate. In the adjusted model we added 2 surrogate variables estimated using `sva` package [2].

In single probe analysis, p-values were corrected using Benjamini and Hochberg (BH) method [3]. A probe was considered as a differentially methylated probe (DMP) if its corrected p-value was smaller than 0.05 (i.e. false discovery rate, FDR<0.05). We run RDA in the target region with 1000 permutations to determine the model p-value. We estimated the R^2 in 1000 random regions to get the probability of finding our results in another region the genome by chance.

We estimated the combined effect of HER2 and ER (Estrogen Receptor) sample subtypes in our target region using RDA. We also run a crude model (with HER2 and ER samples subtypes) and an adjusted model with 2 surrogate variables.

Table S1: Results of expression analysis (40 samples)

Region size	Sim. DEG %	Log FC	R^2 (sd) Target Region	R^2 (sd) Random Region	Recall	Precision
**1Mb**	30	1	0.677 (0.119)	0.027 (0.016)	1	0.935
	30	0.5	0.372 (0.124)	0.026 (0.016)	1	0.939
	10	1	0.421 (0.128)	0.025 (0.015)	1	0.952
	10	0.5	0.178 (0.076)	0.025 (0.015)	0.97	0.946

Values are the mean of the 200 simulations. Sim. DEG %: percentage of differential expressed genes introduced in the simulation. Log FC: Change in gene expression. R^2: R^2 estimate of RDA model. Target region: region that includes our simulated DMPs. Random region: region without any of the simulated DMPs.

Table S2: Results of expression analysis (100 samples)

Region size	Sim. DEG %	Log FC	R^2 (sd) Target Region	R^2 (sd) Random Region	Recall	Precision
**1Mb**	30	1	0.67 (0.119)	0.010 (0.006)	1	0.948
	30	0.5	0.361 (0.12)	0.010 (0.006)	1	0.935
	10	1	0.409 (0.129)	0.011 (0.006)	1	0.948
	10	0.5	0.163 (0.075)	0.010 (0.006)	1	0.948

Values are the mean of the 200 simulations. Sim. DEG %: percentage of differential expressed genes introduced in the simulation. Log FC: Change in gene expression. R^2: R^2 estimate of RDA model. Target region: region that includes our simulated DMPs. Random region: region without any of the simulated DMPs.

Table S3: **Results of expression analysis (10 samples)**

Region size	Sim. DEG %	Log FC	R^2 (sd) Target Region	R^2 (sd) Random Region	Recall	Precision
**1Mb**	30	1	0.718 (0.112)	0.108 (0.060)	0.995	0.948
	30	0.5	0.448 (0.126)	0.113 (0.061)	0.95	0.931
	10	1	0.489 (0.129)	0.109 (0.058)	0.955	0.97
	10	0.5	0.260 (0.091)	0.109 (0.059)	0.675	0.944

Values are the mean of the 200 simulations. Sim. DEG %: percentage of differential expressed genes introduced in the simulation. Log FC: Change in gene expression. R^2: R^2 estimate of RDA model. Target region: region that includes our simulated DMPs. Random region: region without any of the simulated DMPs.

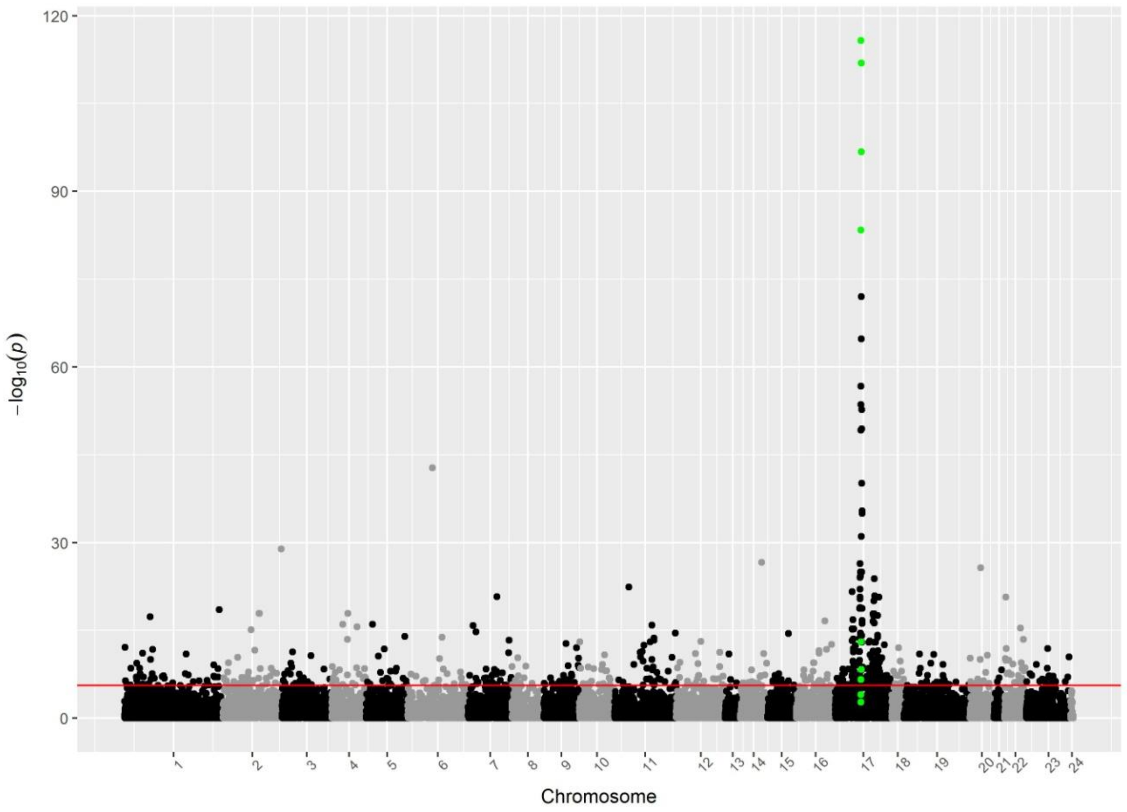

Figure S1: Manhattan plot of the BRCA dataset analysis. These results refer to the statistical significance of the association between each expression probe and the HER2 status. Expression probes are ordered by chromosome and position. Expression probes belonging to the HER2 region are highlighted in green.

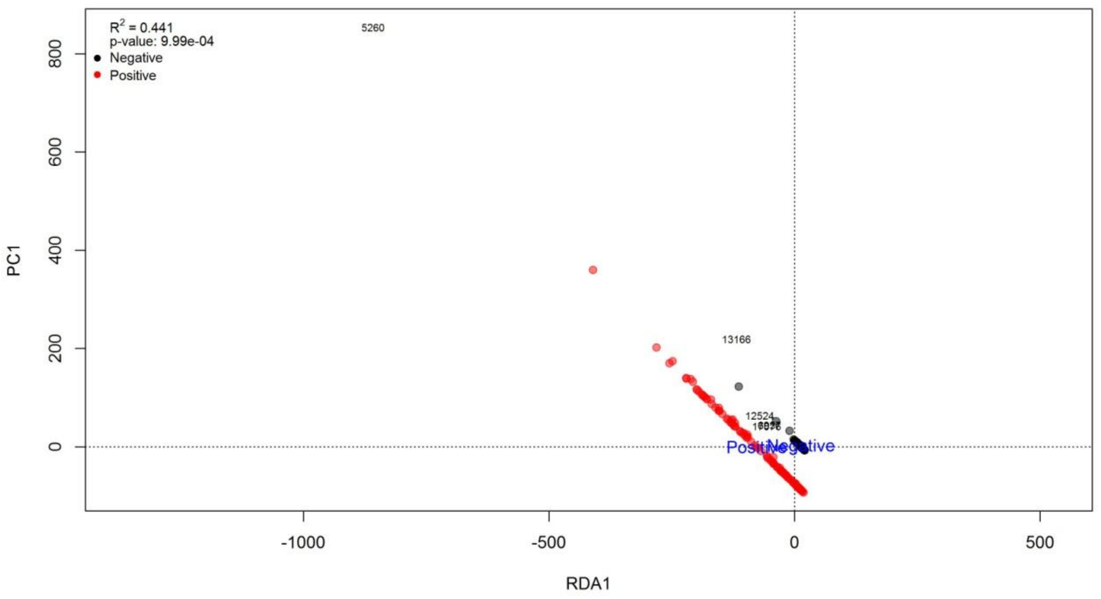

Figure S2: RDA representation of the HER2 region (BRCA dataset). Samples are the points and are coloured depending on the HER2 status (HER2-: Negative, HER2+: Positive). Negative and Positive labels are placed at the centroid of their respective groups. Expression probes most associated with the RDA components are represented with labels. R^2 is the proportion of variance of gene expression data explained by the model. p-value of the RDA's model was computed by sampling the cancer status variable.

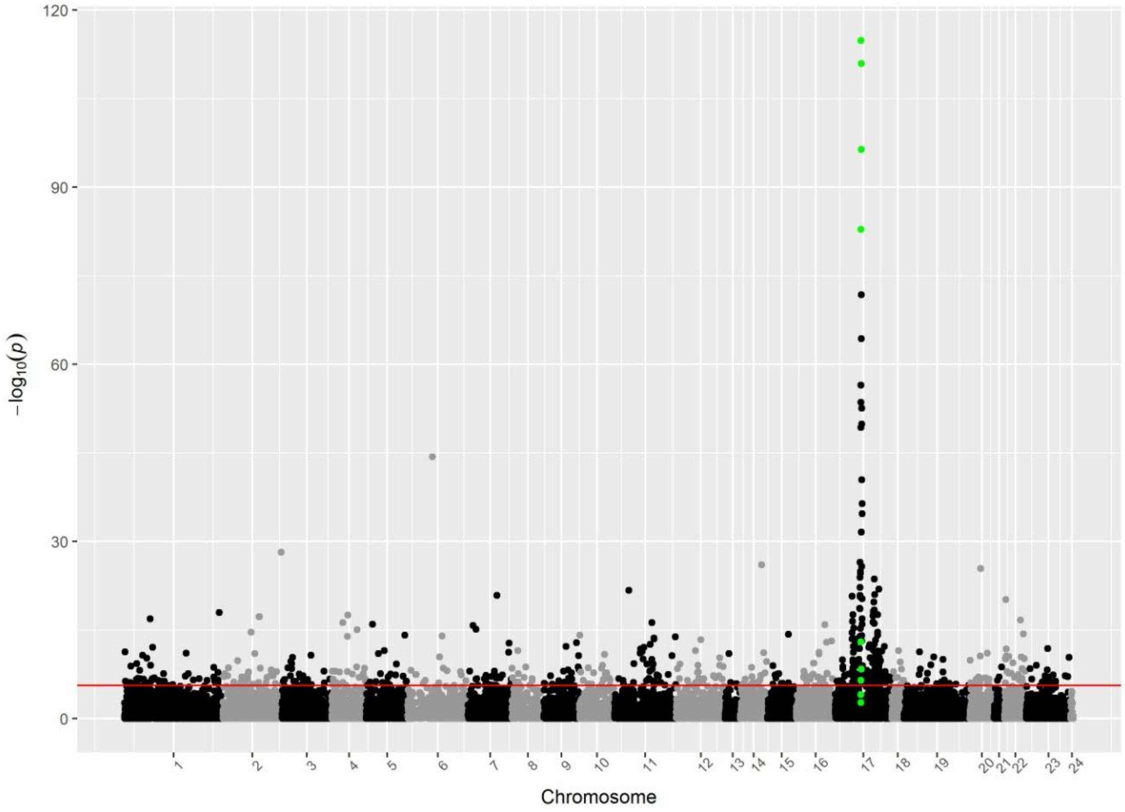

Figure S3: Manhattan plot of the BRCA dataset analysis using adjusted model. These results refer to the statistical significance of the association between each expression probe and the HER2 status. Expression probes are ordered by chromosome and position. Expression probes belonging to the HER2 region are highlighted in green.

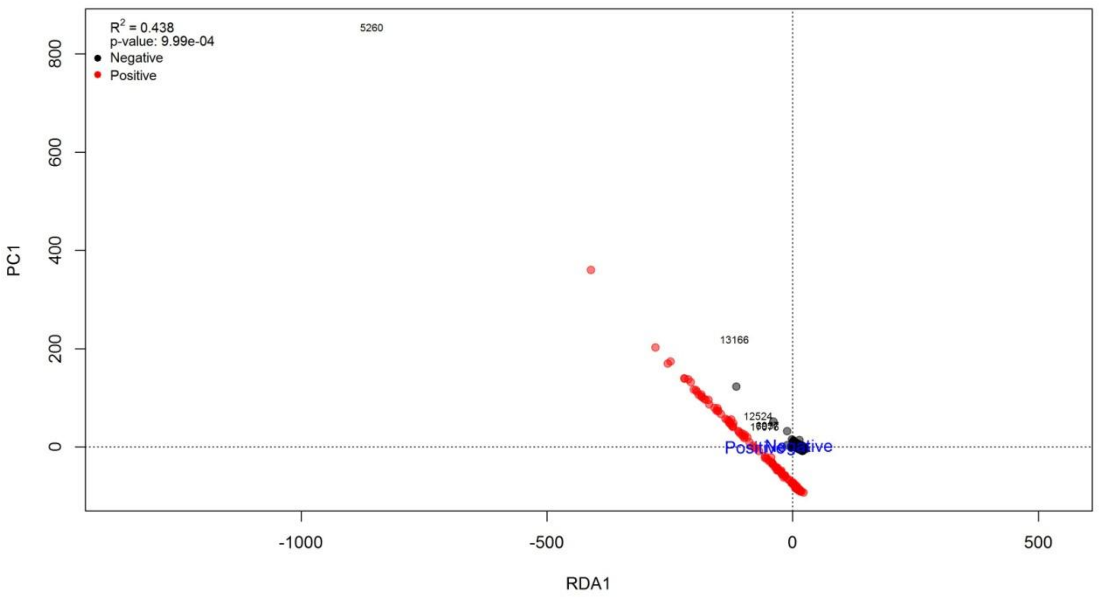

Figure S4: RDA representation of the HER2 region (BRCA dataset) using adjusted model. Samples are the points and are coloured depending on the HER2 status (HER2-: Negative, HER2+: Positive). Negative and Positive labels are placed at the centroid of their respective groups. Expression probes most associated with the RDA components are represented with labels. R^2 is the proportion of variance of gene expression data explained by the model. p-value of the RDA's model was computed by sampling the cancer status variable.

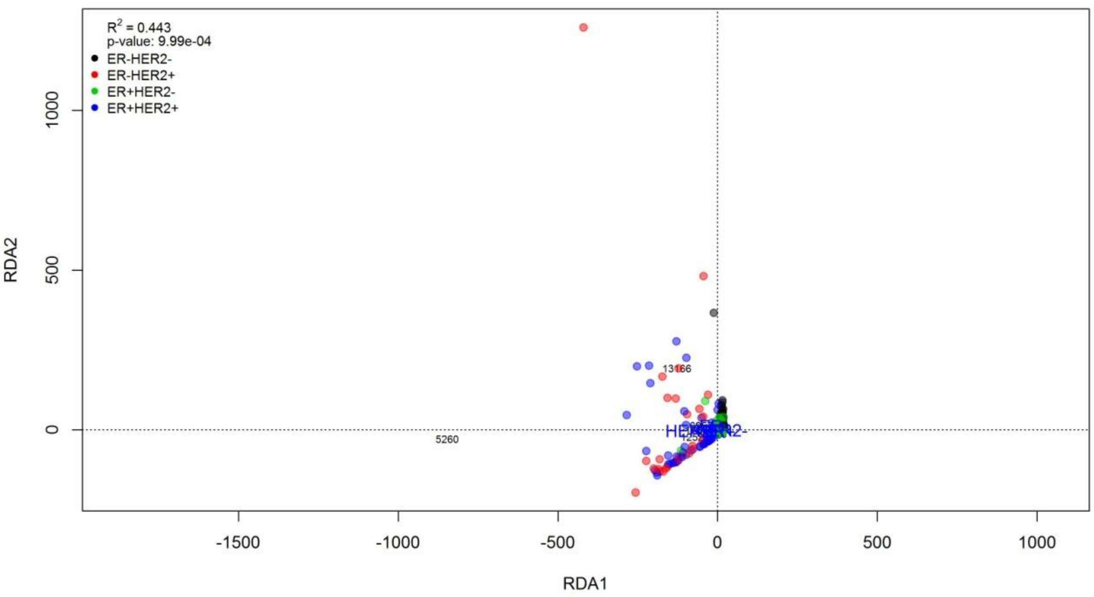

Figure S5: RDA representation of the HER2 region (BRCA dataset) using HER2 and ER sample status. The points are the samples and are coloured depending on the combination of HER2 and ER status. ER-, ER+, HER- and HER+ labels are placed at the centroid of their respective groups. CpGs most associated to the RDA components are represented with labels. R^2 is the proportion of variance of methylation data explained by the model. p-value of the RDA's model was computed by sampling the HER2 and ER sample status variable.

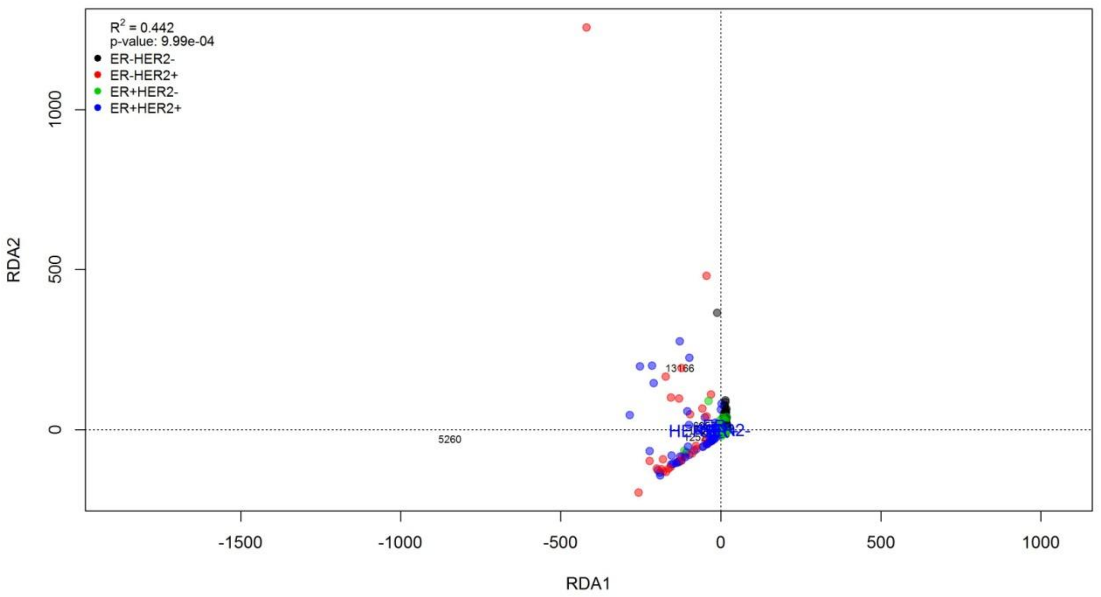

Figure S6: RDA representation of the HER2 region (BRCA dataset) using HER2 and ER sample status (adjusted model). This model also includes surrogate variables. The points are the samples and are coloured depending on the combination of HER2 and ER status. ER-, ER+, HER- and HER+ labels are placed at the centroid of their respective groups. CpGs most associated to the RDA components are represented with labels. R^2 is the proportion of variance of methylation data explained by the model. p-value of the RDA's model was computed by sampling the HER2 and ER sample status variable.

References

1. Colaprico A, Silva TC, Olsen C, Garofano L, Cava C, Garolini D, Sabedot TS, Malta TM, Pagnotta SM, Castiglioni I, Ceccarelli M, Bontempi G, Noushmehr H, T.C.G.A.R. N, M.K. S, P.W. L, G. R, J.N. W, D.J. B, K.A. H, M. C, B.E. B, R.M. M, L. Y, L. C, M. G, T. H, H. N, Y. Z, M.K. S, et al.: ***TCGAbiolinks*** **: an R/Bioconductor package for integrative analysis of TCGA data**. *Nucleic Acids Res* 2016, **44**:e71–e71.

2. Leek J, Johnson W, Parker H, Fertig E, Jaffe A, Storey J: **sva: Surrogate Variable Analysis**. 2016.

3. Benjamini Y, Hochberg Y: **Controlling the False Discovery Rate: A Practical and Powerful Approach to Multiple Testing**. *J R Stat Soc Ser B* 1995, **57**:289–300.
